# Supplementary figures and images for: Effectiveness of accelerated diagnostic protocols for reducing emergency department length of stay in patients presenting with chest pain: A systematic review and meta-analysis
Source: PLoS One. 2024 Oct 22;19(10):e0309767. doi: 10.1371/journal.pone.0309767 (PMC11495623; doi:10.1371/journal.pone.0309767)

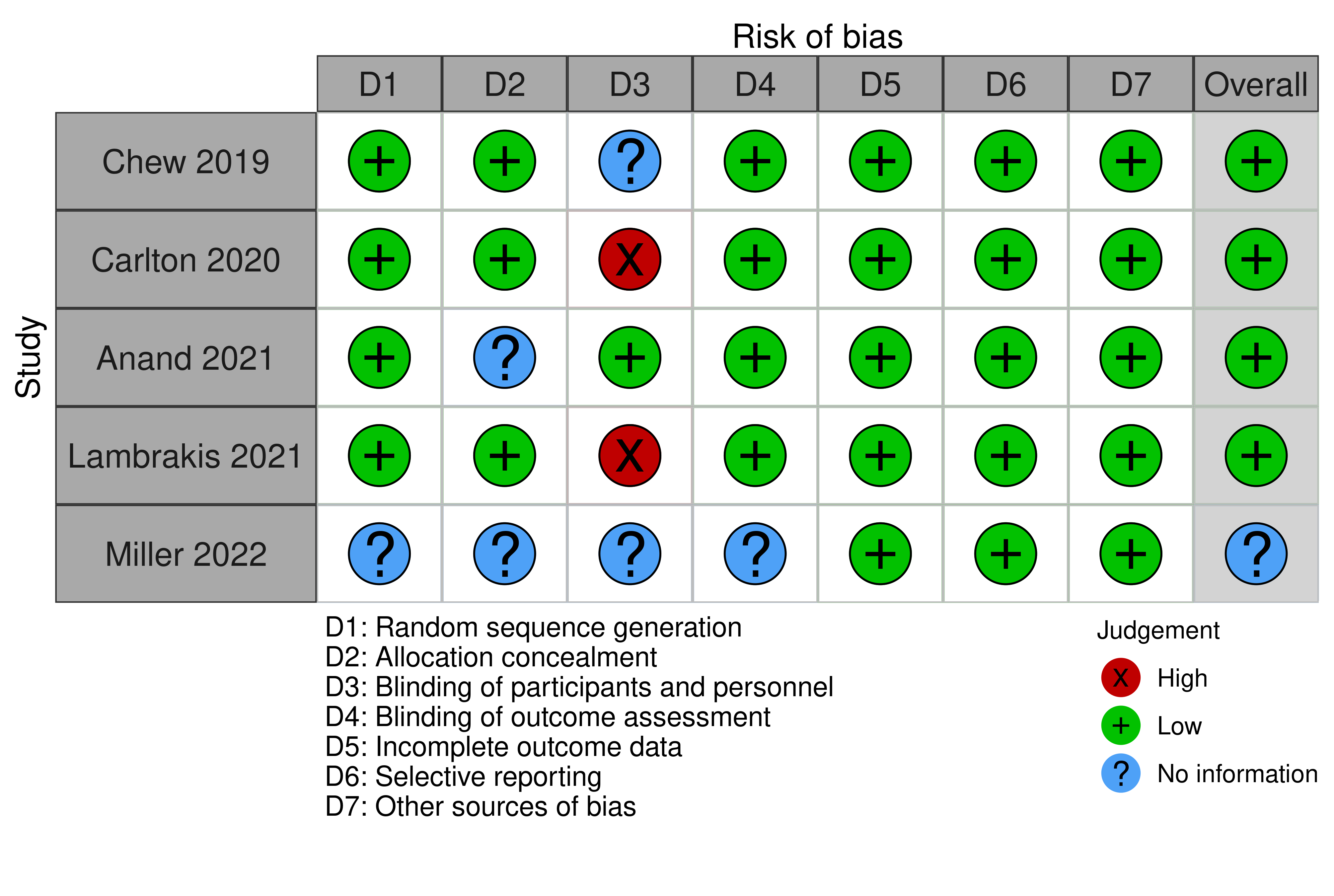

Supplement: S1 Fig — (TIF) [file pone.0309767.s001.tif]

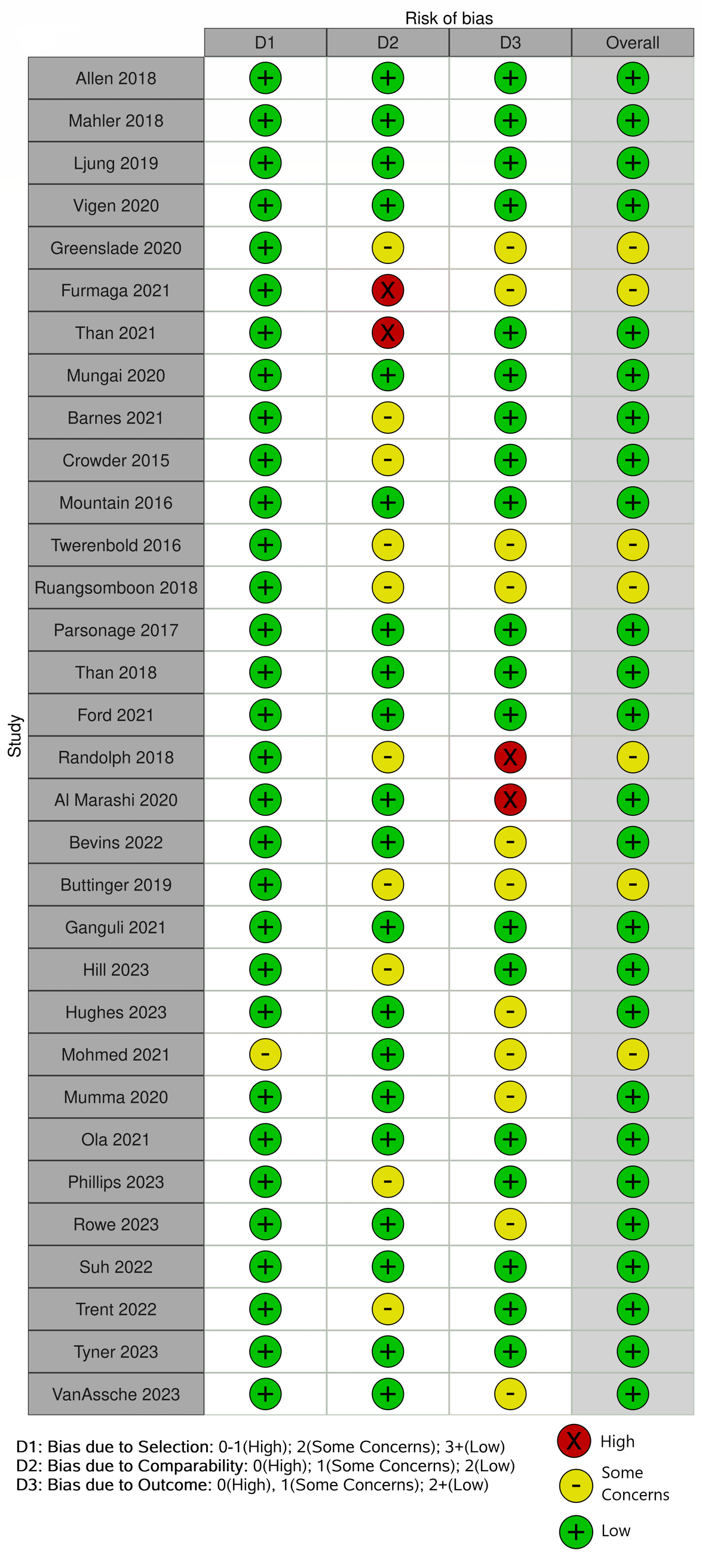

Supplement: S2 Fig — (TIF) [file pone.0309767.s002.tif]

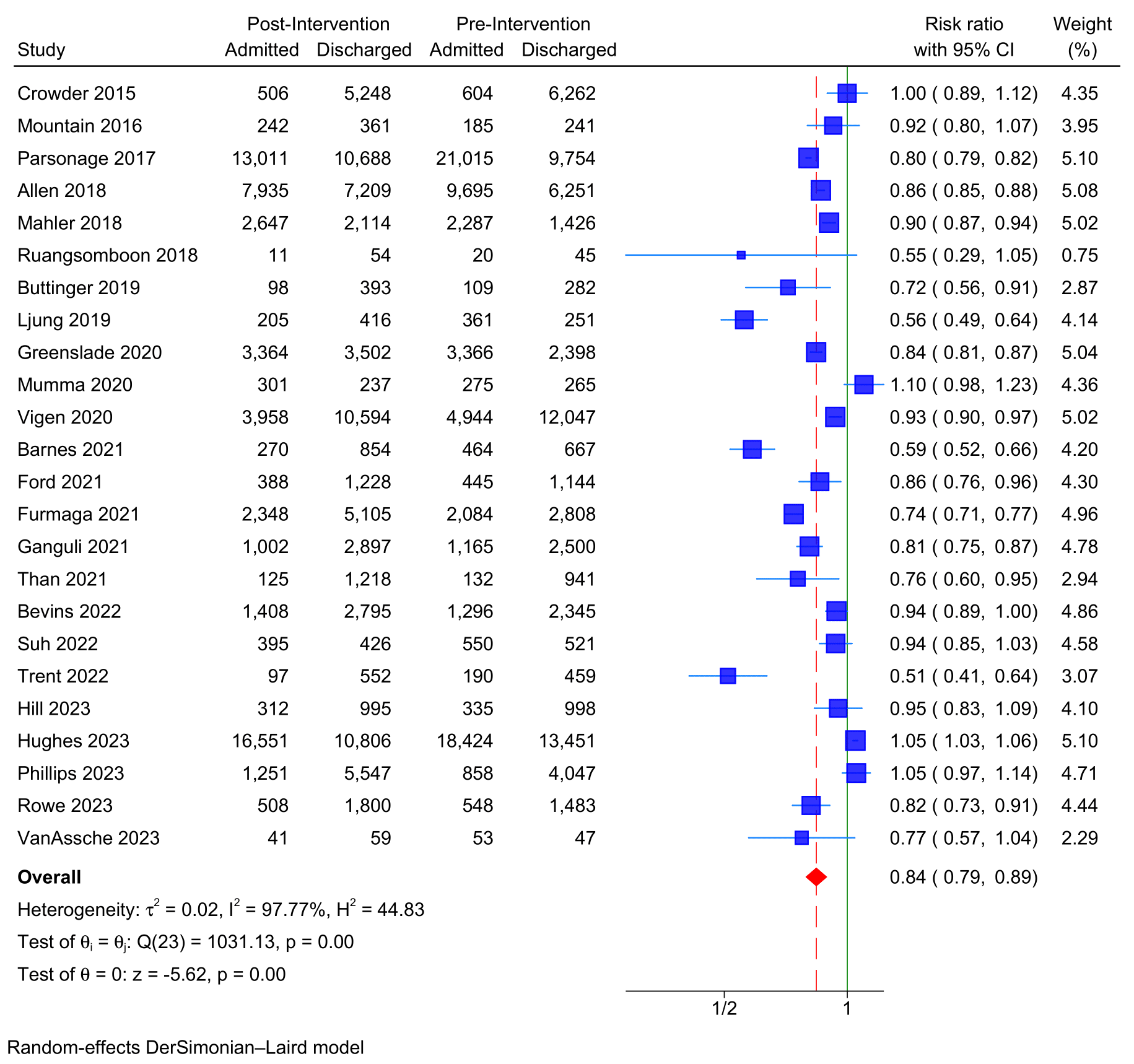

Supplement: S3 Fig — (TIF) [file pone.0309767.s003.tif]

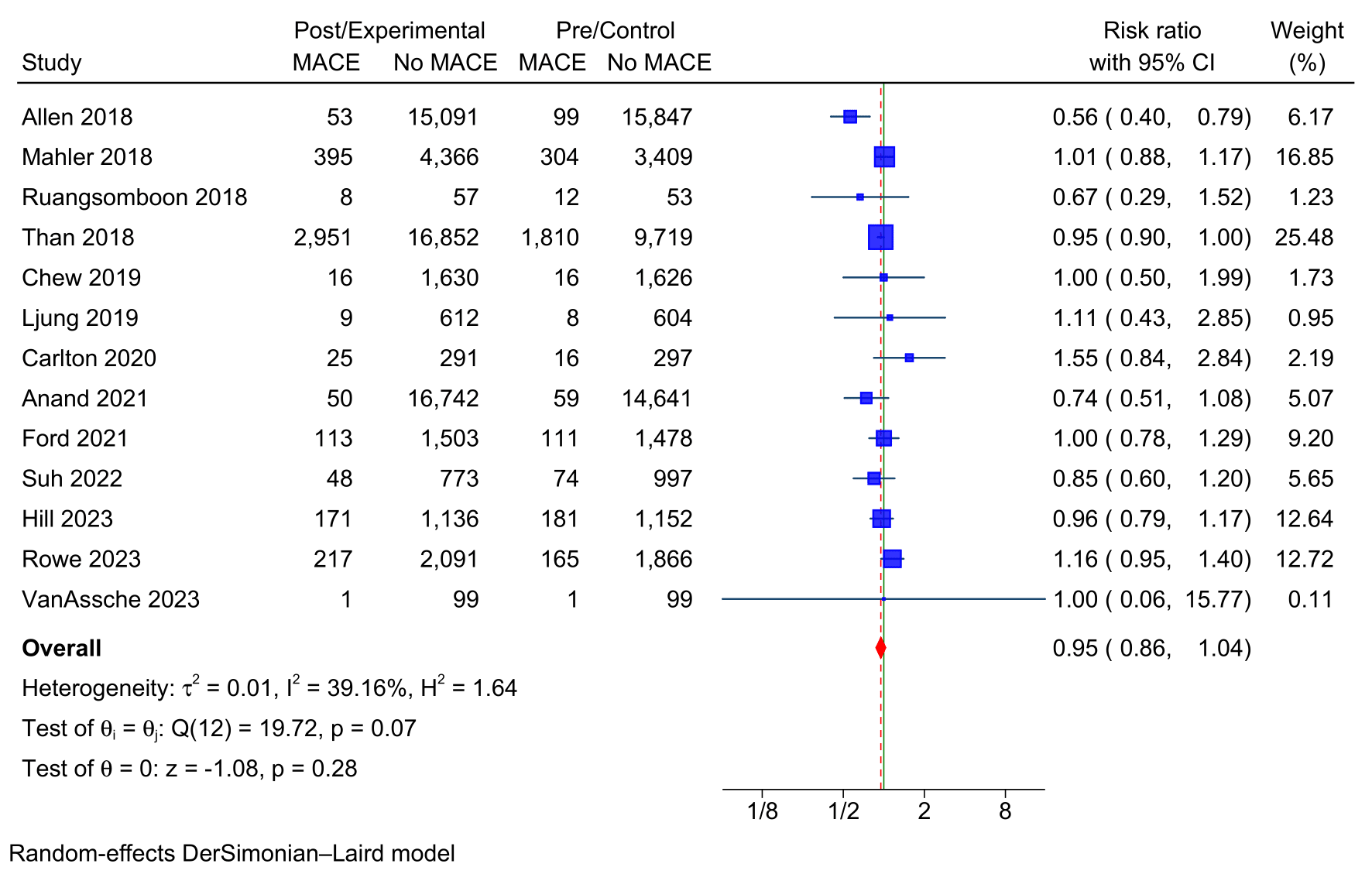

Supplement: S4 Fig — (TIF) [file pone.0309767.s004.tif]
