# Supplementary material for: Effectiveness of accelerated diagnostic protocols for reducing emergency department length of stay in patients presenting with chest pain: A systematic review and meta-analysis
Source: PLoS One. 2024 Oct 22;19(10):e0309767. doi: 10.1371/journal.pone.0309767 (PMC11495623; doi:10.1371/journal.pone.0309767)
Supplement: S1 Table — (DOCX) [file pone.0309767.s005.docx]

| **S1 Table. Proportions of admitted patients and those experiencing MACE (major adverse cardiac events) within 30 days after ADP implementation.** | | | | | | | | |
| --- | --- | --- | --- | --- | --- | --- | --- | --- |
|  |  | **Admissions** | | |  | **MACE** | | |
| **Author** | **Sample size** | **Pre (%)** | **Post (%)** | **Change, % (95% CI)** | ***p*-value** | **Pre (%)** | **Post (%)** | ***p*-value** |
| **Before/After** |  |  |  |  |  |  |  |  |
| Allen | 31,090 | 60.80 | 52.40 | -8.4 (-9.7 to -7.1) | 0.0001 | 0.62 | 0.35 | 0.27 |
| Barnes | 2255 | 41.00 | 24.00 |  | 0.001 |  |  |  |
| Bevins | 7844 | 35.60 | 33.50 |  |  |  |  |  |
| Buttinger | 882 | 27.90 | 20.00 |  |  |  |  |  |
| Crowder | 12620 | 8.80 | 8.80 |  |  |  |  |  |
| Ford | 3205 | 28.00 | 24.00 | -4.0 (-7.3 to -1.3) |  | 7.00 | 7.00 |  |
| Furmaga | 12345 | 42.60 | 31.50 | -11.1 (-15.0 to -7.0) | 0.01 |  |  |  |
| Ganguli | 7564 | 31.80 | 25.70 |  |  |  |  |  |
| Greenslade | 12630 | 58.40 | 49.00 | -9.3 (-11.2 to -7.5) |  |  |  |  |
| Hill | 2640 | 25.10 | 23.90 |  | 0.48 | 13.60 | 13.10 | 0.71 |
| Hughes | 59232 | 57.80 | 60.50 | 2.7 (1.9−3.5) |  |  |  |  |
| Ljung | 1233 | 59.00 | 33.00 | aOR, 0.33 (95% CI, 0.23−0.42) |  | 1.30 | 1.40 |  |
| Mahler | 8474 | 61.60 | 55.60 | -6.0 (-8.1 to -3.9) |  | 8.20 | 8.30 |  |
| Mountain | 1029 | 43.40 | 40.20 |  | 0.07 | 15.20 | 14.30 | 0.768 |
| Mumma | 1078 | 51.00 | 56.00 |  |  |  |  |  |
| Parsonage | 54468 | 68.30 | 54.90 | -13.3 (-18.7 to -8.0) | 0.01 |  |  |  |
| Phillips | 11703 | 17.50 | 18.40 |  | <0.05 |  |  |  |
| Rowe | 4339 | 27.00 | 22.00 | -5.0 (-7.6 to -2.4) |  | 8.10 | 9.40 | 0.14 |
| Ruangsomboon | 130 | 30.80 | 16.90 |  | 0.065 | 19.00 | 12.80 |  |
| Suh | 1892 | 51.40 | 48.10 | -3.3 (-7.9 to 1.2) | 0.150 | 6.90 | 5.80 |  |
| Than | 2416 | 12.30 | 9.30 |  |  |  |  |  |
| Than | 31332 |  |  |  |  | 15.70 | 14.90 |  |
| Trent | 1298 | 29.30 | 14.90 | -14.4 (-18.8 to -10.0) | 0.0001 |  |  |  |
| VanAssche | 200 | 53.00 | 41.00 |  |  | 1.00 | 1.00 | 1.00 |
| Vigen | 31543 | 29.10 | 27.20 |  |  |  |  |  |
|  |  |  |  |  |  |  |  |  |
| **RCTs** |  |  |  |  |  | **Ctrl.** | **Exp.** |  |
| Anand | 31492 |  |  |  |  | 0.40 | 0.30 | 0.068 |
| Carlton | 629 |  |  |  |  | 5.00 | 8.00 |  |
| Chew | 3288 |  |  |  |  | 0.97 | 1.00 | 0.867 |
| ADP, accelerated diagnostic protocol; Ctrl., control group; Exp., experimental group; RCT, randomized controlled trial; MACE, major adverse cardiac events; Pre, before ADP implementation; Post, after ADP implementation. | | | | | | | | |
