# Supplementary material for: Effectiveness of accelerated diagnostic protocols for reducing emergency department length of stay in patients presenting with chest pain: A systematic review and meta-analysis
Source: PLoS One. 2024 Oct 22;19(10):e0309767. doi: 10.1371/journal.pone.0309767 (PMC11495623; doi:10.1371/journal.pone.0309767)
Supplement: S2 Table — (DOCX) [file pone.0309767.s006.docx]

| **S2 Table. Definitions of ED LOS used among the included studies.** | | | | |
| --- | --- | --- | --- | --- |
|  | | | **Type of paper** | **ED LOS definition** |
| Al Marashi | 2020 | Australia | Abstract | ED arrival to ED discharge |
| Allen | 2018 | USA | Full article | ED arrival to ED discharge |
| Barnes | 2021 | Australia | Full article | ED arrival to ED disposition |
| Bevins | 2022 | USA | Full article | Arrival to ED disposition (both discharge and admitted declared separately) |
| Buttinger | 2019 | USA | Abstract | ED arrival to ED discharge |
| Crowder | 2015 | Canada | Full article | ED arrival to ED disposition |
| Ford | 2021 | USA | Full article | ED arrival to ED departure (disposition) |
| Furmaga | 2021 | USA | Full article | ED arrival to ED disposition |
| Ganguli | 2021 | USA | Full article | ED admission to ED discharge |
| Greenslade | 2020 | Australia | Full article | ED arrival to ED disposition |
| Hill | 2023 | Canada | Full article | ED arrival to ED disposition |
| Hughes | 2023 | USA | Full article | ED arrival to ED disposition |
| Ljung | 2019 | Sweden | Full article | ED arrival to ED discharge |
| Mahler | 2018 | USA | Full article | ED arrival to disposition (discharge, transfer, or admission) |
| Mohmed | 2021 | UK | Abstract | ED arrival to disposition |
| Mountain | 2016 | Australia | Full article | ED arrival to ED disposition |
| Mumma | 2020 | USA | Abstract | ED arrival to ED departure (disposition) |
| Mungai | 2020 | USA | Full article | ED arrival to ED disposition |
| Ola | 2021 | USA | Full article | ED arrival to ED disposition |
| Parsonage | 2017 | Australia | Full article | ED arrival to ED disposition |
| Phillips | 2023 | Canada | Full article | ED arrival to disposition (combined, discharged, and admitted reported separately) |
| Randolph | 2018 | USA | Abstract | ED arrival to ED disposition |
| Rowe | 2023 | Canada | Abstract | ED arrival to ED disposition |
| Ruangsomboon | 2018 | Thailand | Full article | ED arrival to ED disposition |
| Suh | 2022 | USA | Full article | First clinician provider evaluation to disposition decision time (provider-to-disposition time, PtDT) |
| Than | 2018 | New Zealand | Full article | ED arrival to ED discharge |
| Than | 2021 | New Zealand | Full article | ED arrival to disposition (discharge also reported) |
| Trent | 2022 | USA | Abstract | ED arrival to disposition (both discharged and admitted reported) |
| Twerenbold | 2016 | Switzerland | Full article | ED arrival to disposition (combined, discharged, and admitted reported separately) |
| Tyner | 2023 | USA | Full article | ED arrival to ED discharge |
| VanAssche | 2023 | Belgium | Full article | ED arrival to ED discharge |
| Vigen | 2020 | USA | Full article | ED dwell time (ED arrival to departure [disposition]) |
| Anand | 2021 | Scotland | Full article | ED arrival (presentation) to hospital discharge |
| Carlton | 2020 | UK | Full article | ED arrival to ED discharge |
| Chew | 2019 | Australia | Full article | ED arrival to ED disposition |
| Lambrakis | 2021 | USA | Full article | ED arrival to ED disposition |
| Miller | 2022 | USA | Abstract | ED arrival to ED disposition |
