# Supplementary material for: Effectiveness of accelerated diagnostic protocols for reducing emergency department length of stay in patients presenting with chest pain: A systematic review and meta-analysis
Source: PLoS One. 2024 Oct 22;19(10):e0309767. doi: 10.1371/journal.pone.0309767 (PMC11495623; doi:10.1371/journal.pone.0309767)
Supplement: S1 File — (DOCX) [file pone.0309767.s007.docx]

**S1 File. Search strategy: Effectiveness of accelerated diagnostic protocols for reducing emergency department length of stay in patients presenting with chest pain: A systematic review**

OVID account EDadp / EDadp1

CITE modified version of Sandy's emergency filter:  Campbell, Sandy.  A Filter to Retrieve Studies related to Emergency Departments from the OVID MEDLINE Database. John W. Scott Health Sciences Library, University of Alberta. Rev. Feb 16, 2016 <http://guides.library.ualberta.ca/ld.php?content_id=14026195>

**Ovid MEDLINE(R) ALL <1946 to October 13, 2023>**

Date searched: Oct 16, 2023

Results: 1233 > 1559

<https://login.ezproxy.library.ualberta.ca/login?url=http://ovidsp.ovid.com/ovidweb.cgi?T=JS&NEWS=N&PAGE=main&SHAREDSEARCHID=3misRcv4dJ2roIrggIZ3WSJXWFRDUjSOf4z48Ec4d5q9zRUd6Bl0F9SEL88azyAua>

1 Chest Pain/ 15126

2 (chest pain or cardiac pain or NSTEMI or non-STEMI or non-ST elevation MI or non-ST elevation myocardial infarction or angina).mp. 116454

3 acute coronary syndrome.mp. 36630

4 1 or 2 or 3 143174

5 ((accelerated or rapid* or early) adj8 (diagnostic protocol* or rule in* or ruling in* or rule out* or ruling out* or pathway or disposition or discharge or (risk adj5 (level or stratif*)) or high risk or low risk)).mp. 42187

6 ((heart adj3 (pathway or protocol)) or (history adj3 age risk factors adj2 troponin adj3 (pathway or protocol)) or EDACS or Emergency department assessment of chest pain score or ADAPT).mp. 70102

7 4 and (5 or 6) 1636

8 ((high* sensitiv* adj2 troponin) or hs-ctn* or hstn* or hs-tn* or hsctn*).mp. 5106

9 (cardiac troponin and (diagnostic protocol* or rule in* or ruling in* or rule out* or ruling out* or disposition or discharge or high risk or low risk)).mp. 1408

10 7 or 8 or 9 7191

11 Emergency Treatment/ or Emergency Medicine/ or emergency medical services/ or emergency service, hospital/ or trauma centers/ or triage/ or exp Evidence-Based Emergency Medicine/ or exp Emergency Nursing/ or Emergencies/ 212694

12 (emergicent* or emergenc* or ED).tw,kf. 545770

13 (triage or casualty department* or ((trauma or urgent) adj2 (cent* or care or facility))).mp. 67214

14 ((accident adj2 emergency) or "a/e" or "a&e").tw,kf. 47373

15 emergen*.jw. 121918

16 or/11-15 730400

17 10 and 16 1740

18 17 not (naturietic or heart failure or cardiac failure or syncope or clinical chemistry score or copeptin).ti. 1633

19 (case reports/ or (case-stud* or case-report*).jw. or (case-study or case series or (case-report not case-report form*)).mp.) not case-control.mp. 2602612

20 18 not 19 1559

**Embase <1974 to 2023 October 13> (OVID Interface)**

Date searched: Oct 16, 2023

Results: 2660 > 3528  (1803 articles and 1455 conf abs)

<https://login.ezproxy.library.ualberta.ca/login?url=http://ovidsp.ovid.com/ovidweb.cgi?T=JS&NEWS=N&PAGE=main&SHAREDSEARCHID=3CWOLo6uOgeJJbNODiEIpbTI3wjVvLueYQtdmBqasb7wZtC9LMsRoerA2yCbo2GPi>

1 thorax pain/ 119801

2 non ST segment elevation myocardial infarction/ 21672

3 (chest pain or cardiac pain or NSTEMI or non-STEMI or non-ST elevation MI or non-ST elevation myocardial infarction or angina or acute coronary syndrome).mp. 257369

4 ((accelerated or rapid* or early) adj8 (diagnostic protocol* or rule in* or ruling in* or rule out* or ruling out* or pathway or disposition or discharge or (risk adj5 (level or stratif*)) or high risk or low risk)).mp. 64904

5 ((heart adj3 (pathway or protocol)) or (history adj3 age risk factors adj2 troponin adj3 (pathway or protocol)) or EDACS or Emergency department assessment of chest pain score or ADAPT).mp. 87339

6 (1 or 2 or 3) and (4 or 5) 3449

7 ((high* sensitiv* adj2 troponin) or hs-ctn* or hstn* or hs-tn* or hsctn*).mp. 10287

8 (cardiac troponin and (diagnostic protocol* or rule in* or ruling in* or rule out* or ruling out* or disposition or discharge or high risk or low risk)).mp. 2888

9 6 or 7 or 8 14513

10 emergency treatment/ or emergency medicine/ or exp emergency health service/ or evidence based emergency medicine/ or emergency nursing/ or exp emergency care/ or emergency ward/ or emergency/ or (emergicent* or emergenc* or ED or (accident adj2 emergency) or "a/e" or "a&e").tw,kw. or (triage or casualty department* or ((trauma or urgent) adj2 (cent* or care or facility))).mp. or emergen*.jx. 1057390

11 9 and 10 3929

12 11 not (naturietic or heart failure or cardiac failure or syncope or clinical chemistry score or copeptin).ti. 3689

13 (Case report/ or (case-stud* or case-report*).jx. or (case-study or case series or (case-report not case-report-form*)).mp.) not case-control.mp. 3188956

14 12 not 13 3258

**Cochrane library (Trials database only) (WILEY interface)**

Date searched: October 16, 2023

Results: 258  >308

Search saved as: Chest pain accelerated diagnostic protocols in ldennett account

#1 [mh ^"Chest Pain"] or (chest-pain or cardiac-pain or NSTEMI or non-STEMI or non-ST-elevation-MI or non-ST-elevation-myocardial-infarction or angina or acute-coronary-syndrome):ti,ab,kw

#2 ((accelerated or rapid* or early) near/8 (diagnostic-protocol or rule-in or ruling-in or rule-out or ruling-out or pathway or disposition or discharge or (risk near/5 (level or stratif*)) or high-risk or low-risk)):ti,ab,kw OR ((heart near/3 (pathway or protocol)) or (history near/3 age-risk-factors near/2 troponin near/3 (pathway or protocol)) or EDACS or Emergency-department-assessment-of-chest-pain-score or ADAPT):ti,ab,kw

#3 ((high* sensitiv* near/2 troponin) or hs-ctn* or hstn* or hs-tn* or hsctn*):ti,ab,kw or (cardiac troponin and (diagnostic protocol* or rule in* or ruling in* or rule out* or ruling out* or disposition or discharge or high risk or low risk)):ti,ab,kw

#4 [mh ^"Emergency Treatment"] or [mh ^"Emergency Medicine"] or [mh ^"emergency medical services"] or [mh ^"emergency service, hospital"] or [mh ^"trauma centers"] or [mh ^"triage"] or [mh "Evidence-Based Emergency Medicine"] or [mh "Emergency Nursing"] or [mh ^"Emergencies"] or (emergicent* or emergenc* or casualty-department or ED or triage or ((trauma or urgent) near/2 (cent* or care or facility)) or (accident near/2 emergency) or "a/e" or "a&e"):ti,ab,kw or emergen*:so

#5 ((#1 AND #2) OR #3) AND #4

#6 #5 not (naturietic or heart-failure or cardiac-failure or syncope or clinical-chemistry-score or copeptin):ti

**SCOPUS (Advanced Search)**

Date searched: October 16, 2023

Results: 1516 > 1952

( ( ( TITLE-ABS-KEY ( chest-pain  OR  cardiac-pain  OR  nstemi  OR  non-stemi  OR  non-st-elevation-mi  OR  non-st-elevation-myocardial-infarction  OR  angina  OR  acute-coronary-syndrome )  AND  TITLE-ABS-KEY ( ( ( accelerated  OR  rapid*  OR  early )  W/8  ( diagnostic-protocol*  OR  rule-in*  OR  ruling-in*  OR  rule-out*  OR  ruling-out*  OR  pathway  OR  disposition  OR  discharge  OR  ( risk  W/5  ( level  OR  stratif* ) )  OR  high-risk  OR  low-risk ) )  OR  ( heart  W/3  ( pathway  OR  protocol ) )  OR  ( history  W/3  age-risk-factors  W/2  troponin  W/3  ( pathway  OR  protocol ) )  OR  edacs  OR  emergency-department-assessment-of-chest-pain-score  OR  {ADAPT} ) )  OR  TITLE-ABS-KEY ( ( high*-sensitiv*  W/2  troponin )  OR  hs-ctn*  OR  hstn*  OR  hs-tn*  OR  hsctn*  OR  ( cardiac-troponin  AND  ( diagnostic-protocol*  OR  rule-in*  OR  ruling-in*  OR  rule-out*  OR  ruling-out*  OR  disposition  OR  discharge  OR  high-risk  OR  low-risk ) ) ) )  AND  ( TITLE-ABS-KEY ( "a/e"  OR  "a&e"  OR  emergicent*  OR  emergenc*  OR  "ED"  OR  ( accident  W/2  emergency )  OR  triage  OR  casualty-department*  OR  ( ( trauma  OR  urgent )  W/2  ( cent*  OR  care  OR  facility ) ) )  OR  SRCTITLE ( emergenc* ) ) )  AND NOT  ( TITLE ( naturietic  OR  heart-failure  OR  cardiac-failure  OR  syncope  OR  clinical-chemistry-score  OR  copeptin )  OR  ( SRCTITLE ( case-stud*  OR  case-report* )  OR  TITLE-ABS-KEY ( {case study}  OR  {case report}  OR  {case-series} ) )  AND NOT  TITLE-ABS-KEY ( {case-control} ) )

**CINAHL Plus with Full Text (EBSCOhost Interface)**

Date searched: April 23, 2021

Results: 878 > 798

Deselect "Apply equivalent subjects"

Search saved as: Chest pain accelerated diagnostic protocols - CINAHL in ldennett account

S1. (MH "Chest Pain+") or chest-pain or cardiac-pain or NSTEMI or non-STEMI or non-ST-elevation-MI or non-ST-elevation-myocardial-infarction or angina or acute-coronary-syndrome   40,596

S2. ((accelerated or rapid* or early) N8 (diagnostic protocol* or rule-in* or ruling-in* or rule-out* or ruling-out* or pathway or disposition or discharge or (risk N5 (level or stratif*)) or high-risk or low-risk)) OR (heart N3 (pathway or protocol)) or (history N3 age-risk-factors N2 troponin N3 (pathway or protocol)) or EDACS or Emergency-department-assessment-of-chest-pain-score or "ADAPT"    29,940

S3. (high* sensitiv* N2 troponin) or hs-ctn* or hstn* or hs-tn* or hsctn* or (cardiac-troponin and (diagnostic protocol* or rule-in* or ruling-in* or rule-out* or ruling-out* or disposition or discharge or high-risk or low-risk))   2,273

S4.  (MH "Emergency Service+") or (MH "Emergency Medicine") or (MH "Physicians, Emergency") OR (MH "Emergency Nurse Practitioners") or (MH "Emergency Nursing+") or "a/e" or "a&e" or emergicent* or emergenc* or "ED" or (accident N2 emergency) or triage or casualty-department* or ((trauma or urgent) N2 (cent* or care or facility)) or SO(emergenc*)  349,709

S5. ((S1 AND S2) OR S3) AND S4  846

S6. S5 NOT (TI(naturietic or heart-failure or cardiac-failure or syncope or clinical-chemistry-score or copeptin) OR (((MH "Case Studies") OR SO(case-stud* or case-report*) OR ( "case study" or "case report" or "case series") ) NOT (case-control)))   798

**Dissertations and Theses Global (Proquest Interface)**

Date searched: Oct 15, 2023

Results: 4 >  14

( ( noft( chest-pain  OR  cardiac-pain  OR  nstemi  OR  non-stemi  OR  non-st-elevation-mi  OR  non-st-elevation-myocardial-infarction  OR  angina  OR  acute-coronary-syndrome )  AND  noft(noft) ( ( ( noft(accelerated)  OR  noft(rapid*)  OR  noft(early) )  NEAR/8  ( noft(diagnostic-protocol)  OR  noft(rule-in*)  OR  noft(ruling-in*)  OR  noft(rule-out*)  OR  noft(ruling-out*)  OR  noft(pathway)  OR  noft(disposition)  OR  noft(discharge)  OR  ( noft(risk)  NEAR/5  ( noft(level)  OR  noft(stratif*) ) )  OR  noft(high-risk)  OR  noft(low-risk) ) )  OR  ( noft(heart)  NEAR/3  ( noft(pathway)  OR  noft(protocol) ) )  OR  ( noft(history)  NEAR/3  noft(age-risk-factors)  NEAR/2  noft(troponin)  NEAR/3  ( noft(pathway)  OR  noft(protocol) ) )  OR  noft(edacs)  OR  noft(emergency-department-assessment-of-chest-pain-score)  OR  noft("ADAPT") ) )  OR  noft( ( high*-sensitiv*  NEAR/2  troponin )  OR  hs-ctn*  OR  hstn*  OR  hs-tn*  OR  hsctn*  OR  ( cardiac-troponin  AND  ( diagnostic-protocol*  OR  rule-in*  OR  ruling-in*  OR  rule-out*  OR  ruling-out*  OR  disposition  OR  discharge  OR  high-risk  OR  low-risk ) ) ) )  AND  ( noft( "a/e"  OR  "a&e"  OR  emergicent*  OR  emergenc*  OR  "ED"  OR  ( accident  NEAR/2  emergency )  OR  triage  OR  casualty-department*  OR  ( ( trauma  OR  urgent )  NEAR/2  ( cent*  OR  care  OR  facility ) ) )  )

**LILACS https://lilacs.bvsalud.org/en/**

Date searched: Oct 15, 2023

Downloaded 16 results

**chest-pain emergency-department high-sensitivity-troponin (8 results)**

**chest-pain emergency-department high-sensitivity-cardiac-troponin (3 duplicate results)**

**chest-pain emergency-department accelerated-diagnostic-protocol (1 additional results)**

**chest-pain emergency-department HEART-protocol (8 results, 1 of these is duplicate)**

**chest-pain emergency-department HEART-pathway (1 duplicate results)**

**chest-pain emergency-department EDACS (1 duplicate results)**

**Google scholar (searched via Publish or Perish)**

Date searched: Oct 15, 2023

Results: Downloaded first 200

chest-pain emergency-department high-sensitivity-troponin OR high-sensitivity-cardiac-troponin OR hs-ctn* OR or OR hstn* OR hs-tn* OR hsctn* OR accelerated-diagnostic-protocol* OR HEART-protocol OR HEART-pathway OR EDACS OR ADAPT-protocol OR ADAPT-pathway
