# Supplementary material for: Effectiveness of accelerated diagnostic protocols for reducing emergency department length of stay in patients presenting with chest pain: A systematic review and meta-analysis
Source: PLoS One. 2024 Oct 22;19(10):e0309767. doi: 10.1371/journal.pone.0309767 (PMC11495623; doi:10.1371/journal.pone.0309767)
Supplement: S3 File — (DOCX) [file pone.0309767.s009.docx]

| **S3 File. Characteristics of excluded studies (ordered by study ID)** | | |
| --- | --- | --- |
| **Reference no.** | **Study** | **Reason for exclusion** |
| [1] | Agrawal 2018 | No outcome of interest |
| [2] | Aldous 2012a | Data duplication |
| [3] | Aldous 2012b | No outcome of interest |
| [4] | Aldous 2012c | No outcome of interest |
| [5] | Aldous 2012d | No outcome of interest |
| [6] | Aldous 2017 | Inappropriate patient population |
| [7] | Alfonso 2016 | No outcome of interest |
| [8] | Allen 2021 | No outcome of interest |
| [9] | Ambavane 2017 | Data duplication |
| [10] | Andruchow 2020 | No outcome of interest |
| [11] | Badertscher 2018 | No outcome of interest |
| [12] | Bahrmann 2013 | No outcome of interest |
| [13] | Bandstein 2017a | No outcome of interest |
| [14] | Bandstein 2017b | No outcome of interest |
| [15] | Baugh 2016 | Inappropriate study setting |
| [16] | Baugh 2019 | No outcome of interest |
| [17] | Bellini 2021 | No outcome of interest |
| [18] | Biener 2013 | No outcome of interest |
| [19] | Body 2016 | Data duplication |
| [20] | Boeddinghaus 2017 | No outcome of interest |
| [21] | Boeddinghaus 2018 | No outcome of interest |
| [22] | Borna 2018 | No outcome of interest |
| [23] | Bove 2017 | No outcome of interest |
| [24] | Bracco 2010 | No outcome of interest |
| [25] | Braga 2011 | No outcome of interest |
| [26] | Breckner 2017 | No outcome of interest |
| [27] | Bularga 2019 | No outcome of interest |
| [28] | Bunch 2016 | Inappropriate intervention |
| [29] | Burgio 2018 | No outcome of interest |
| [30] | Burgstaller 2020 | No outcome of interest |
| [31] | Bylund 2021 | cTn also used in post-group |
| [32] | Carlton 2015 | Data duplication |
| [33] | Carlton 2016 | Data duplication |
| [34] | Chapman 2017 | No outcome of interest |
| [35] | Chapman 2018 | No outcome of interest |
| [36] | Chuang 2021 | Data duplication |
| [37] | Chuang 2022 | Data duplication |
| [38] | Collinson 2017 | No outcome of interest |
| [39] | Conde 2013 | No outcome of interest |
| [40] | Cook 2023 | Data duplication |
| [41] | Corsini 2015 | No outcome of interest |
| [42] | Cortes 2018 | No outcome of interest |
| [43] | Costabel 2014 | No outcome of interest |
| [44] | Costabel 2019 | No outcome of interest |
| [45] | Croce 2017 | No outcome of interest |
| [46] | Crowder 2012 | Data duplication |
| [47] | Crowder 2014 | Data duplication |
| [48] | Cullen 2013 | Data duplication |
| [49] | Cullen 2014 | Data duplication |
| [50] | Cullen 2022 | Inappropriate intervention |
| [51] | Dadkhah 2015 | cTn also used in post-group |
| [52] | Dongxu 2021 | No outcome of interest |
| [53] | Fabbri 2019 | Inappropriate intervention |
| [54] | Fesmire 2002 | Inappropriate intervention |
| [55] | Ford 2020 | Data duplication |
| [56] | Frisoli 2017 | Inappropriate intervention |
| [57] | Gafni-Pappas 2018 | No outcome of interest |
| [58] | George 2013 | No outcome of interest |
| [59] | Greenslade 2013 | Data duplication |
| [60] | Greenslade 2020 | Data duplication |
| [61] | Halder 2021 | Inappropriate intervention |
| [62] | Hammarsten 2017 | No outcome of interest |
| [63] | Hammerer-Lercher 2013 | No outcome of interest |
| [64] | Heidari 2021 | cTn also used in post-group |
| [65] | Hill 2023 | cTn also used in post-group |
| [66] | Hricak 2016 | Intervention not mentioned |
| [67] | Hyams 2018 | No outcome of interest |
| [68] | Johannessen 2020 | Inappropriate study setting |
| [69] | Johannessen 2022 | Inappropriate study setting |
| [70] | Julicher 2017 | Data duplication |
| [71] | Kaambwa 2017 | Inappropriate intervention |
| [72] | Kashef 2018 | No intervention |
| [73] | Keller 2011 | No outcome of interest |
| [74] | Khan 2022 | Inappropriate intervention |
| [75] | Kienbacher 2021 | No outcome of interest |
| [76] | Lateef 2001 | No intervention |
| [77] | Lau 2020 | No intervention |
| [78] | Leahu 2021 | No intervention |
| [79] | Lin 2021 | No outcome of interest |
| [80] | Lin 2022 | Inappropriate outcome |
| [81] | Lippi 2017 | Inappropriate intervention |
| [82] | Lund 2005 | Inappropriate intervention |
| [83] | Mahler 2015 | Data duplication |
| [84] | Mahler 2016 | Study protocol |
| [85] | Marcusohn 2020 | No intervention |
| [86] | Marjot 2019 | No outcome of interest |
| [87] | Mark 2021 | No intervention |
| [88] | Meller 2015 | Inappropriate intervention |
| [89] | Milburn 2016a | Data duplication |
| [90] | Milburn 2016b | Data duplication |
| [91] | Miller 2022 | Data duplication |
| [92] | Mokhtari 2016 | No outcome of interest |
| [93] | Mokhtari 2017 | No outcome of interest |
| [94] | Mueller 2016 | No outcome of interest |
| [95] | Munro 2015 | No outcome of interest |
| [96] | Nagree 2015 | Intervention not mentioned |
| [97] | Nestelberger 2019 | No outcome of interest |
| [98] | Neumann 2016 | No outcome of interest |
| [99] | Ng 2001 | cTn also used in post-group |
| [100] | Nilsson 2021 | No outcome of interest |
| [101] | Nowak 2018 | cTn also used in post-group |
| [102] | Nowak 2020 | No outcome of interest |
| [103] | O’Rielly 2020 | Data duplication |
| [104] | Paoloni 2010 | No intervention |
| [105] | Parsonage 2014 | Data duplication |
| [106] | Parsonage 2016 | Data duplication |
| [107] | Peacock 2020 | No outcome of interest |
| [108] | Peck 2016 | Inappropriate intervention |
| [109] | Perera 2018 | No outcome of interest |
| [110] | Pettersson 2018 | No outcome of interest |
| [111] | Pickering 2015 | No outcome of interest |
| [112] | Pickering 2016 | Data duplication |
| [113] | Pickering 2018 | Data duplication |
| [114] | Poldervaart 2016 | No outcome of interest |
| [115] | Poldervaart 2017 | No outcome of interest |
| [116] | Rabbani 2003 | No outcome of interest |
| [117] | Rao 2012 | No outcome of interest |
| [118] | Reichlin 2012 | Data duplication |
| [119] | Reichlin 2015a | Data duplication |
| [120] | Reichlin 2015b | No outcome of interest |
| [121] | Richards 2017 | No outcome of interest |
| [122] | Rottger 2017 | No outcome of interest |
| [123] | Ruangsomboon 2021 | No outcome of interest |
| [124] | Sanchis 2014 | No outcome of interest |
| [125] | Sandoval 2017a | Inappropriate outcome |
| [126] | Sandoval 2017b | Inappropriate outcome |
| [127] | Sandoval 2020 | No outcome of interest |
| [128] | Schonemann-Lund 2015 | No outcome of interest |
| [129] | Shah 2015 | Inappropriate intervention |
| [130] | Shah 2018 | Inappropriate intervention |
| [131] | Sharp 2019 | No outcome of interest |
| [132] | Shiozaki 2017 | No outcome of interest |
| [133] | Shiozaki 2020 | No outcome of interest |
| [134] | Shortt 2017 | No outcome of interest |
| [135] | Skoien 2015 | Intervention not mentioned |
| [136] | Slagman 2017 | Intervention not mentioned |
| [137] | Smulowitz 2018 | No outcome of interest |
| [138] | Snavely 2021 | No outcome of interest |
| [139] | Stopyra 2016 | Data duplication |
| [140] | Stopyra 2020a | No outcome of interest |
| [141] | Stopyra 2020b | No outcome of interest |
| [142] | Stopyra 2021 | Inappropriate patient population |
| [143] | Storrow 2015 | No intervention |
| [144] | Stoyanov 2019a | Inappropriate study setting |
| [145] | Stoyanov 2019b | Inappropriate study setting |
| [146] | Stoyanov 2020a | Inappropriate outcome |
| [147] | Stoyanov 2020b | Inappropriate outcome |
| [148] | Tan 2019 | Inappropriate outcome |
| [149] | Than 2011 | Inappropriate outcome |
| [150] | Than 2012 | No outcome of interest |
| [151] | Than 2014a | No outcome of interest |
| [152] | Than 2014b | No outcome of interest |
| [153] | Than 2016 | No outcome of interest |
| [154] | Thet 2019 | No outcome of interest |
| [155] | Thiruganasambandamoorthy 2020 | Inappropriate outcome |
| [156] | Twerenbold 2014 | Data duplication |
| [157] | Twerenbold 2017 | Data duplication |
| [158] | Twerenbold 2018a | Data duplication |
| [159] | Twerenbold 2018b | Data duplication |
| [160] | Twerenbold 2019 | Inappropriate outcome |
| [161] | Whitehead 2017 | Inappropriate intervention |
| [162] | Wildi 2016 | Data duplication |
| [163] | Wildi 2017 | No outcome of interest |
| [164] | Yang 2020 | No outcome of interest |
| [165] | Yau 2017 | No outcome of interest |
| [166] | Yip 2014 | No outcome of interest |

**References**

1. Agrawal AV, Shrivastava R, Singh M. Validation of 0 -2 hour algorithm for rule in and rule out myocardial infarction based on highly sensitive troponin I assay. Indian Heart J. 2018;70:S41‐2.

2. Aldous SJ, Richards M, Cullen L, Troughton R, Than M. A 2-hour thrombolysis in myocardial infarction score outperforms other risk stratification tools in patients presenting with possible acute coronary syndromes: comparison of chest pain risk stratification tools. Am Heart J. 2012;164:516-23.

3. Aldous SJ, Richards M, Cullen L, Troughton R, Than M. Diagnostic and prognostic utility of early measurement with high-sensitivity troponin T assay in patients presenting with chest pain. CMAJ. 2012;184:E260-8.

4. Aldous S, Pemberton C, Richards AM, Troughton R, Than M. High-sensitivity troponin T for early rule-out of myocardial infarction in recent onset chest pain. Emerg Med J. 2012;29:805-10.

5. Aldous SJ, Richards MA, Cullen L, Troughton R, Than M. A new improved accelerated diagnostic protocol safely identifies low-risk patients with chest pain in the emergency department. Acad Emerg Med. 2012;19:510-516.

6. Aldous S. Improving care processes for patients with possible suspected acute coronary syndrome (Icare-ACS): A national implementation trial of a clinical guidance framework. Heart Lung Circ. 2017;26:S3.

7. Alfonso F, Salamanca J, Del Pozo E. High-sensitivity troponin testing in the diagnosis of acute coronary syndrome in the emergency department. Emergencias. 2016;28:283-4.

8. Allen BR, Christenson RH, Cohen SA, Nowak R, Wilkerson RG, Mumma B, et al. Diagnostic Performance of High Sensitivity Cardiac Troponin T Strategies and Clinical Variables in a Multisite United States Cohort. Circulation. 2021;143(17):1659-72.

9. Ambavane A, Lindahl B, Giannitsis E, Roiz J, Mendivil J, Frankenstein L, et al. Economic evaluation of the one-hour rule-out and rule-in algorithm for acute myocardial infarction using the high-sensitivity cardiac troponin T assay in the emergency department. PLoS One. 2017;12:e0187662.

10. Andruchow JE, Boyne T, Seiden-Long I, Wang D, Vatanpour S, Innes G, et al. Prospective comparative evaluation of the European Society of Cardiology (ESC) 1-hour and a 2-hour rapid diagnostic algorithm for myocardial infarction using high-sensitivity troponin-T. CJEM. 2020;22:712-20.

11. Badertscher P, Boeddinghaus J, Twerenbold R, Nestelberger T, Wildi K, Wussler D, et al. Direct Comparison of the 0/1h and 0/3h Algorithms for Early Rule-Out of Acute Myocardial Infarction. Circulation. 2018;137(23):2536-8.

12. Bahrmann P, Christ M, Bahrmann A, Rittger H, Heppner HJ, Achenbach S, et al. A 3-hour diagnostic algorithm for non-ST-elevation myocardial infarction using high-sensitivity cardiac troponin T in unselected older patients presenting to the emergency department. J Am Med Dir Assoc. 2013;14:409-16.

13. Bandstein N, Wikman A, Ljung R, Holzmann MJ. Survival and resource utilization in patients with chest pain evaluated with cardiac troponin T compared with high-sensitivity cardiac troponin T. Int J Cardiol. 2017;245:43-8.

14. Bandstein N, Ljung R, Lundback M, Johansson M, Holzmann MJ. Trends in admissions for chest pain after the introduction of high-sensitivity cardiac troponin T. Int J Cardiol. 2017;240:1-7.

15. Baugh CW, Greenberg JO, Mahler SA, Kosowsky JM, Schuur JD, Parmar S, et al. Implementation of a Risk Stratification and Management Pathway for Acute Chest Pain in the Emergency Department. Crit Pathw Cardiol. 2016;15(4):131-7.

16. Baugh CW, Scirica BM, Januzzi JL, Morrow DA, Lewandrowski KB, Jarolim P, et al. Implementation of an Emergency Department High-Sensitivity Troponin Chest Pain Pathway in the United States. Crit Pathw Cardiol. 2019;18:1-4.

17. Bellini C, Cinci F, Bova G, Mascarucci M, Leoncini R, Scapellato C, et al. Methodology to Evaluate Clinical Impact of 0/3 Hour High-Sensitivity Cardiac Troponin T Protocol on Managing Acute Coronary Syndrome in Daily Emergency Department Practice. Lab Med. 2021;52(5):452-9.

18. Biener M. Comparison of a 3-hour versus a 6-hour sampling-protocol using high-sensitivity cardiac troponin T for rule-out and rule-in of non-STEMI in an unselected emergency department population. Int J Cardiol. 2013;167:1134-40.

19. Body R, Mueller C, Giannitsis E, Christ M, Ordonez-Llanos J, de Filippi CR, et al. The Use of Very Low Concentrations of High-sensitivity Troponin T to Rule Out Acute Myocardial Infarction Using a Single Blood Test. Acad Emerg Med. 2016;23:1004-13.

20. Boeddinghaus J, Nestelberger T, Twerenbold R, Wildi K, Badertscher P, Cupa J, et al. Direct Comparison of 4 Very Early Rule-Out Strategies for Acute Myocardial Infarction Using High-Sensitivity Cardiac Troponin I. Circulation. 2017;135:1597-11.

21. Boeddinghaus J, Nestelberger T, Twerenbold R, Neumann JT, Lindahl B, Giannitsis E, et al. Impact of age on the performance of the ESC 0/1h-algorithms for early diagnosis of myocardial infarction. Eur Heart J. 2018;39:3780-94.

22. Borna C, Kollberg K, Larsson D, Mokhtari A, Ekelund U. The objective CORE score allows early rule out in acute chest pain patients. Scand Cardiovasc J. 2018;52:308-14.

23. Bove J, Hochman S, Miller J, Artim S. Effectiveness of 2-hour Troponin in High-risk Patients With Suspected Acute Coronary Syndrome. Crit Pathw Cardiol. 2017;16:53-7.

24. Bracco C, Melchio R, Sturlese U, Pomero F, Martini G, Poggi A, et al. Early stratification of patients with chest pain and suspected acute coronary syndrome in the Emergency Department. Minerva Med. 2010;101:73-80.

25. Braga F, Dolci A, Valente C, Boido A, Moretti A, Guzzetti S, et al. Impact of the implementation of highly sensitive cardiac troponin T assay in a university hospital setting. Biochim Clin. 2011;35:301-6.

26. Breckner G, Walker J, Hanley K, Butki N. Utilizing PDSA Cycle in Implementing a Chest Pain Accelerated Diagnostic Protocol. Spartan Med Res J. 2017;2:6436.

27. Bularga A, Lee KK, Stewart S, Ferry AV, Chapman AR, Marshall L, et al. High-Sensitivity Troponin and the Application of Risk Stratification Thresholds in Patients with Suspected Acute Coronary Syndrome. Circulation. 2019;140:1557-68.

28. Bunch AM, Leasure AR, Carithers C, Burnette RE, Berryman MS. Implementation of a rapid chest pain protocol in the emergency department: A quality improvement project. J Am Assoc Nurse Pract. 2016;28:75-83.

29. Burgio MA, Marino G. cTnT-hs in the early diagnosis of acute myocardial infarction: evaluation of Rapid rule-out (0-1 h) in an Emergency Department population. Rivista Italiana della Medicina di Laboratorio. 2018;14:208-15.

30. Burgstaller JM, Held U, Gravestock I, Klauser BS, Gort LM, Melzer L, et al. Impact of the Introduction of High-Sensitive Troponin Assay in the Emergency Department: A Retrospective Study. Am J Med. 2020;133:976-85.

31. Bylund WE, Cole PM, Lloyd ML, Mercer AA, Osit AK, Hussain SW, et al. Effect of Implementation of HEART Chest Pain Protocol on Emergency Department Disposition, Testing and Cost. West J Emerg Med. 2021;22:308-18.

32. Carlton EW, Cullen L, Than M, Gamble J, Khattab A, Greaves K. A novel diagnostic protocol to identify patients suitable for discharge after a single high-sensitivity troponin. Heart. 2015;101:1041-6.

33. Carlton EW. A novel accelerated diagnostic protocol to identify emergency department patients with chest pain who may be suitable for discharge after a single high-sensitivity troponin: trust (triage rule-out using high-sensitivity troponin) chest pain study. PQDT - UK & Ireland. 2016.

34. Chapman AR, Anand A, Boeddinghaus J, Ferry A V, Sandeman D, Adamson PD, et al. Comparison of the Efficacy and Safety of Early Rule-Out Pathways for Acute Myocardial Infarction. Circulation. 2017;135:1586-96.

35. Chapman AR, Hesse K, Andrews J, Lee KK, Anand A, Shah AS V, et al. High-Sensitivity Cardiac Troponin I and Clinical Risk Scores in Patients With Suspected Acute Coronary Syndrome. Circulation. 2018;138:1654-65.

36. Chuang A, Gnanamanickam E, Lambrakis K, Horsfall M, Karnon J, Blyth A, et al. Cost effectiveness of a 1-hour high-sensitivity troponin-t protocol in suspected acute coronary syndrome: A trialbased analysis of the rapid-tnt randomised trial. Circulation. 2021;144:S6984.

37. Chuang M-YA, Gnanamanickam ES, Karnon J, Lambrakis K, Horsfall M, Blyth A, et al. Cost effectiveness of a 1-hour high-sensitivity troponin-T protocol: An analysis of the RAPID-TnT trial. Int J Cardiol Heart Vasc. 2022;38:100933.

38. Collinson PO, Gaze D, Goodacre S. Evaluation of the European society of cardiology recommended rapid diagnostic algorithms in a challenging low risk cohort. Eur Heart J. 2017;63:S25.

39. Conde D, Costabel JP, Lambardi F. Algorithm for probable acute coronary syndrome using high-sensitivity troponin T assay vs fourth-generation troponin T assay. Am J Emerg Med. 2013;31:1226-9.

40. Cook B, Gandolfo C, Klausner HA, McCord JM, Miller JB, Mills N, et al. Results of a Cluster Randomized Trial Implementing a Rapid High-Sensitivity Troponin I Protocol. J Emerg Med. 2023;64:419-20.

41. Corsini A, Vagnarelli F, Bugani G, Bacchi Reggiani ML, Semprini F, Nanni S, et al. Impact of high-sensitivity Troponin T on hospital admission, resources utilization, and outcomes. Eur Heart J Acute Cardiovasc Care. 2015;4:148-57.

42. Cortes MM, Lambardi F, Ariznavarreta P, Resi S, Arbucci R, Borda M, et al. Usefulness of the HEART score with high-sensitivity troponin T for the evaluation of patients with chest pain. Rev Argent Cardiol. 2018;86:317-21.

43. Costabel JP, Lambardi F, Barboza AC, Cobo AL, Aragon M, Conde D, et al. Evaluation of a new diagnostic algorithm for acute coronary syndrome using high-sensitivity troponin T assay. Rev Argent Cardiol. 2014;82:298-303.

44. Costabel JP, Ariznavarreta P, Lambardi F, Arbucci R, Vergara JM, Katib C, et al. Resultados de los primeros pacientes con sospecha de síndrome coronario agudo evaluados con el algoritmo de 1 hora propuesto por la Sociedad Europea de Cardiología. Rev Argent Cardiol. 2019;87:197-202.

45. Croce A, Brunati P, Colzani C, Terramocci R, Favero S, Bordoni G, et al. A Rational Adoption of the High Sensitive Assay for Cardiac Troponin I in Diagnostic Routine. Dis Markers. 2017;2017:4523096.

46. Crowder K, Jones T, Wang D, Clark S, Innes G, Lang E, et al. The effect of implementing high-sensitivity troponin testing on operational efficiency in three large urban emergency departments. Can J Cardiol. 2012;28:S135.

47. Crowder K, Jones T, Wang D, Steve C, Jamie M, James A, et al. Operational impact and patient outcomes following implementation of high-sensitivity troponin testing in three urban emergency departments. Ann Emerg Med. 2014;64:S110.

48. Cullen L, Mueller C, Parsonage WA, Wildi K, Greenslade JH, Twerenbold R, et al. Validation of high-sensitivity troponin I in a 2-hour diagnostic strategy to assess 30-day outcomes in emergency department patients with possible acute coronary syndrome. J Am Coll Cardiol. 2013;62:1242-49.

49. Cullen L, Greenslade JH, Than M, Brown AFT, Hammett CJ, Lamanna A, et al. The new Vancouver Chest Pain Rule using troponin as the only biomarker: an external validation study. Am J Emerg Med. 2014;32:129-34.

50. Cullen L, Stephensen L, Greenslade J, Starmer K, Starmer G, Stone R, et al. Emergency Department Assessment of Suspected Acute Coronary Syndrome Using the IMPACT Pathway in Aboriginal and Torres Strait Islander People. Heart Lung Circ. 2022;31:1029-36.

51. Dadkhah S, Almuwaqqat Z, Rawal HA, Sulaiman S, Husein H, Elhangouche N, et al. Evaluation of chest pain in the emergency room using a 2 hour sensitive troponin I (cTnI). J Am Col Cardiol. 2015;65:A149.

52. Dongxu C, Yannan Z, Yilin Y, Chenling Y, Guorong G, Dongwei S, et al. Evaluation of the 0 h/1 h high-sensitivity cardiac troponin T algorithm in diagnosis of non-ST-segment elevation myocardial infarction (NSTEMI) in Han population. Clin Chem Lab Med. 2021;59:757-64.

53. Fabbri A, Bachetti C, Ottani F, Morelli A, Benazzi B, Spiezia S, et al. Rapid rule-out of suspected acute coronary syndrome in the Emergency Department by high-sensitivity cardiac troponin T levels at presentation. Intern Emerg Med. 2019;14:403-10.

54. Fesmire FM, Hughes AD, Fody EP, Jackson AP, Fesmire CE, Gilbert MA, et al. The Erlanger chest pain evaluation protocol: a one-year experience with serial 12-lead ECG monitoring, two-hour delta serum marker measurements, and selective nuclear stress testing to identify and exclude acute coronary syndromes. Ann Emerg Med. 2002;40:584-94.

55. Ford J, Chaco E, Tancredi DJ, Mumma BE. High-sensitivity cardiac troponin and emergency department length of stay, admission rate, and myocardial infarction diagnoses. Acad Emerg Med. 2020;27:S89.

56. Frisoli TM, Nowak R, Evans KL, Harrison M, Alani M, Varghese S, et al. Henry Ford HEART Score Randomized Trial: Rapid Discharge of Patients Evaluated for Possible Myocardial Infarction. Circ Cardiovasc Qual Outcomes. 2017;10(10):e003617.

57. Gafni-Pappas G, DeMeester SD, Boyd MA, Ganti A, Nicholson AM, Albright J, et al. The HAS-Choice study: Utilizing the HEART score, an ADP, and shared decision-making to decrease admissions in chest pain patients. Am J Emerg Med. 2018;36:1825-31.

58. George T, Ashover S, Cullen L, Larsen P, Gibson J, Bilesky J, et al. Introduction of an accelerated diagnostic protocol in the assessment of emergency department patients with possible acute coronary syndrome: the Nambour Short Low-Intermediate Chest pain project. Emerg Med Australas. 2013;25:340-4.

59. Greenslade JH, Cullen L, Than M, Aldous S, Chu K, Brown AFT, et al. Validation of the Vancouver Chest Pain Rule using troponin as the only biomarker: a prospective cohort study. Am J Emerg Med. 2013;31:1103-7.

60. Greenslade JH, Parsonage W, Foran L, McCormack L, Ashover S, Milburn T, et al. Widespread Introduction of a High-Sensitivity Troponin Assay: Assessing the Impact on Patients and Health Services. J Clin Med. 2020;9:1883.

61. Halder D, Mathew R, Jamshed N, Yadav S, Rl B, Aggarwal P, et al. Utility of HEART Pathway in Identifying Low-Risk Chest Pain in Emergency Department. J Emerg Med. 2021;60(4):421-7.

62. Hammarsten O, Bjurman C, Holzmann M, Lindahl B. Troponinnivåer ger nu bättre hjälp vid misstänkt hjärtinfarkt - Låga nivåer kan med hög säkerhet utesluta hjärtinfarkt – nya analysmetoder ökar den medicinska säkerheten. Lakartidningen. 2017;114.

63. Hammerer-Lercher A, Ploner T, Neururer S, Schratzberger P, Griesmacher A, Pachinger O, et al. High-sensitivity cardiac troponin T compared with standard troponin T testing on emergency department admission: how much does it add in everyday clinical practice? J Am Heart Assoc. 2013;2:e000204.

64. Heidari K, Arani MA, Sheibani M, Pickering JW, Chouhdari A. Comparison of two diagnostic protocols in the management of possible cardiac chest pain: One follow-up study in Iran. Caspian J Intern Med. 2021;12:148-54.

65. Hill J, Yang E, Lefebvre D, Doran S, Raizman J, Tsui A, et al. An accelerated diagnostic protocol for patients with cardiac chest pain using conventional troponin I on emergency department length of stay: a single site retrospective cohort study. Can J Emerg Med. 2023;25:S73-4.

66. Hricak V, Kamensky G, Margoczy R, Danova K, Murin J. Where is the place of serum high sensitivity troponin T in the early diagnostic of acute myocardial infarction? Cardiol Lett. 2016;25:79-84.

67. Hyams JM, Streitz MJ, Oliver JJ, Wood RM, Maksimenko YM, Long B, et al. Impact of the HEART Pathway on Admission Rates for Emergency Department Patients with Chest Pain: An External Clinical Validation Study. J Emerg Med. 2018;54:549-57.

68. Johannessen TR, Vallersnes OM, Halvorsen S, Larstorp ACK, Mdala I, Atar D. Pre-hospital One-Hour Troponin in a Low-Prevalence Population of Acute Coronary Syndrome: OUT-ACS study. Open Heart. 2020;7(2):e001296.

69. Johannessen TR, Halvorsen S, Atar D, Munkhaugen J, Nore AK, Wisloff T, et al. Cost-effectiveness of a rule-out algorithm of acute myocardial infarction in low-risk patients: emergency primary care versus hospital setting. BMC Health Serv Res. 2022;22:1274.

70. Julicher P, Greenslade JH, Parsonage WA, Cullen L. The organisational value of diagnostic strategies using high-sensitivity troponin for patients with possible acute coronary syndromes: a trial-based cost-effectiveness analysis. BMJ Open. 2017;7:e013653.

71. Kaambwa B, Ratcliffe J, Horsfall M, Astley C, Karnon J, Coates P, et al. Cost effectiveness of high-sensitivity troponin compared to conventional troponin among patients presenting with undifferentiated chest pain: A trial based analysis. Int J Cardiol. 2017;238:144-50.

72. Kashef MA, Garb J, Kugelmass A, Lotfi A. Implementation of an Early Discharge Protocol and Chest Pain Clinic for Low-Risk Chest Pain in the Emergency Department. Crit Pathw Cardiol. 2018;17:1-5.

73. Keller T, Zeller T, Ojeda F, Tzikas S, Lillpopp L, Sinning C, et al. Serial changes in highly sensitive troponin I assay and early diagnosis of myocardial infarction. JAMA. 2011;306:2684-93.

74. Khan E, Lambrakis K, Nazir SA, Chuang A, Halabi A, Tiver K, et al. Implementation of more sensitive cardiac troponin T assay in a state-wide health service. Int J Cardiol. 2022;347:66-72.

75. Kienbacher CL, Fuhrmann V, van Tulder R, Havel C, Schreiber W, Rasoul-Rockenschaub S, et al. Impact of more conservative European Society of Cardiology guidelines on the management of patients with acute chest pain. Int J Clin Pract. 2021;75(6):e14133.

76. Lateef F, Storrow AB, Malone K, Liu T, Gibler BW. Comparison of a 6-hour and 9-hour protocol for evaluation of moderate-to-low risk chest pain patients in an emergency department diagnostic unit. Singapore Med J. 2001;42:52-6.

77. Lau G, Koh M, Kavsak PA, Schull MJ, Armstrong DWJ, Udell JA, et al. Clinical outcomes for chest pain patients discharged home from emergency departments using high-sensitivity versus conventional cardiac troponin assays. Am Heart J. 2020;221:84-94.

78. Leahu AI, Mion MM, Padoan A, Zaninotto M, Plebani M. High-sensitive method to measure troponin I and efficiency: The results of an experience. Biochim Clin. 2021;44:S54-8.

79. Lin Z, Lim SH, Yap QV, Tan CHC, Chan YH, Wong HC, et al. Comparing conventional and high sensitivity troponin T measurements in identifying adverse cardiac events in patients admitted to an Asian emergency department chest pain observation unit. Int J Cardiol Heart Vasc. 2021;34:100758.

80. Lin Z, Cardelli P, Marino R, Lim SH, Di Somma S. Advantage of Using of High-Sensitivity Troponin I Compared to Conventional Troponin I in Shortening Time to Rule out/in Acute Coronary Syndrome in Chest Pain Patients Presenting to the Emergency Department. Medicina (Kaunas). 2022;58:1391.

81. Lippi G, Bonfanti L, Dipalo M, Aloe R, Cervellin G. Clinical, organizational and economic analysis of high-sensitivity cardiac troponin testing in the emergency department. Ann Res Hosp. 2017;1:14.

82. Lund J, Ilva T, Porela P, Eriksson S, Pettersson K, Mustonen H, et al. Improved early risk stratification and diagnosis of myocardial infarction, using a novel troponin I assay concept. Eur J Clin Invest. 2005;35:112-6.

83. Mahler SA, Riley RF, Hiestand BC, Russell GB, Hoekstra JW, Lefebvre CW, et al. The HEART Pathway randomized trial: identifying emergency department patients with acute chest pain for early discharge. Circ Cardiovasc Qual Outcomes. 2015;8:195-203.

84. Mahler SA, Burke GL, Duncan PW, Case LD, Herrington DM, Riley RF, et al. HEART Pathway Accelerated Diagnostic Protocol Implementation: Prospective Pre-Post Interrupted Time Series Design and Methods. JMIR Res Protoc. 2016;5:e10.

85. Marcusohn E, Epstein D, Roguin A, Zukermann R. Rapid rule out for suspected myocardial infarction: is the algorithm appropriate for all?. Eur Heart J Qual Care Clin Outcomes. 2020;6:193-8.

86. Marjot J, Kaier TE, Henderson K, Hunter L, Marber MS, Perera D. A single centre prospective cohort study addressing the effect of a rule-in/rule-out troponin algorithm on routine clinical practice. Eur Heart J Acute Cardiovasc Care. 2019;8:404-11.

87. Mark DG, Huang J, Kene M V, Sax DR, Cotton DM, Lin JS, et al. Prospective Validation and Comparative Analysis of Coronary Risk Stratification Strategies Among Emergency Department Patients With Chest Pain. J Am Heart Assoc. 2021;10:e020082.

88. Meller B, Cullen L, Parsonage WA, Greenslade JH, Aldous S, Reichlin T, et al. Accelerated diagnostic protocol using high-sensitivity cardiac troponin T in acute chest pain patients. Int J Cardiol. 2015;184:208-15.

89. Milburn T, Ashover S, Skoien W, Cullen L, Greenslade J, Parsonage W. A large scale implementation of the adapt accelerated diagnostic protocol into clinical practice in Queensland: Impact on hospital length of stay and admission rates for possible cardiac chest pain. Heart Lung Circ. 2016;25:S6-7.

90. Milburn T, Ashover S, Skoien W, Parsonage W, Greenslade J, Bright S, et al. Economic impact of the accelerated chest pain risk evaluation (ACRE) project-A large scale implementation of the adapt accelerated diagnostic protocol into clinical practice. Heart Lung Circ. 2016;25:S43.

91. Miller J, Gunaga S, Krupp S, Klausner H, Plemmons E, Nasseredine H, et al. 49 Comparing the Safety and Efficacy of a Rapid High-Sensitivity Cardiac Troponin I Protocol Between Hospital-Based and Free-Standing Emergency Departments. Ann Emerg Med. 2022;80:S26.

92. Mokhtari A, Borna C, Gilje P, Tyden P, Lindahl B, Nilsson H-J, et al. A 1-h Combination Algorithm Allows Fast Rule-Out and Rule-In of Major Adverse Cardiac Events. J Am Coll Cardiol. 2016;67:1531-40.

93. Mokhtari A, Lindahl B, Schiopu A, Yndigegn T, Khoshnood A, Gilje P, et al. A 0-Hour/1-Hour Protocol for Safe, Early Discharge of Chest Pain Patients. Acad Emerg Med. 2017;24:983-92.

94. Mueller C, Giannitsis E, Christ M, Ordonez-Llanos J, deFilippi C, McCord J, et al. Multicenter Evaluation of a 0-Hour/1-Hour Algorithm in the Diagnosis of Myocardial Infarction With High-Sensitivity Cardiac Troponin T. Ann Emerg Med. 2016;68:76-87.e4.

95. Munro AR, Jerram T, Morton T, Hamilton S. Use of an Accelerated Diagnostic Pathway allows rapid and safe discharge of 70% of chest pain patients from the emergency department. N Z Med J. 2015;128:62-71.

96. Nagree Y. A rapid chest pain pathway that reduced ED length of stay by 20%. Emerg Med Australas. 2015;27:18.

97. Nestelberger T, Boeddinghaus J, Greenslade J, Parsonage WA, Than M, Wussler D, et al. Two-Hour Algorithm for Rapid Triage of Suspected Acute Myocardial Infarction Using a High-Sensitivity Cardiac Troponin I Assay. Clin Chem. 2019;65:1437-47.

98. Neumann JT, Sorensen NA, Schwemer T, Ojeda F, Bourry R, Sciacca V, et al. Diagnosis of Myocardial Infarction Using a High-Sensitivity Troponin I 1-Hour Algorithm. JAMA Cardiol. 2016;1:397-404.

99. Ng SM, Krishnaswamy P, Morissey R, Clopton P, Fitzgerald R, Maisel AS. Ninety-minute accelerated critical pathway for chest pain evaluation. Am J Cardiol. 2001;88:611-7.

100. Nilsson T, Johannesson E, Lundager Forberg J, Mokhtari A, Ekelund U. Diagnostic accuracy of the HEART Pathway and EDACS-ADP when combined with a 0-hour/1-hour hs-cTnT protocol for assessment of acute chest pain patients. Emerg Med J. 2021;38:808-13.

101. Nowak RM, Gandolfo CM, Jacobsen G, Christenson RH, Moyer M, Hudson M, et al. Ultrarapid Rule-out for Acute Myocardial Infarction Using the Generation 5 Cardiac Troponin T Assay: Results From the REACTION-US Study. Ann Emerg Med. 2018;72:654-64.

102. Nowak RM, Christenson RH, Jacobsen G, McCord J, Apple FS, Singer AJ, et al. Performance of Novel High-Sensitivity Cardiac Troponin I Assays for 0/1-Hour and 0/2- to 3-Hour Evaluations for Acute Myocardial Infarction: Results From the HIGH-US Study. Ann Emerg Med. 2020;76:1-13.

103. O’Rielly C, Andruchow J, McRae A. LO17: Major adverse cardiac events in patients ruled-out by a validated high-sensitivity troponin algorithm for acute myocardial infarction. Canadian Association of Emergency Physicians (CAEP/ACMU) Conference, June 1-4, 2020, Ontario, Canada. Can J Emerg Med. 2020;22:S13.

104. Paoloni R, Kumar P, Janu M. Pilot study of high-sensitivity troponin T testing to facilitate safe early disposition decisions in patients presenting to the emergency department with chest pain. Intern Med J. 2010;40:188-92.

105. Parsonage WA, Greenslade JH, Hammett CJ, Lamanna A, Tate JR, Ungerer JP, et al. Validation of an accelerated high-sensitivity troponin T assay protocol in an Australian cohort with chest pain. Med J Aust. 2014;200:161-5.

106. Parsonage WA, Ashover S, Milburn T, Skoien W, Greenslade J, Cullen L. Translation of the ADAPT accelerated diagnostic protocol into clinical practice: Impact on hospital length of stay and admission rates for possible cardiac chest pain. Eur Heart J. 2016;37:228-9.

107. Peacock WF, Christenson R, Diercks DB, Fromm C, Headden GF, Hogan CJ, et al. Myocardial Infarction Can Be Safely Excluded by High-sensitivity Troponin I Testing 3 Hours After Emergency Department Presentation. Acad Emerg Med. 2020;27:671-80.

108. Peck D, Knott J, Lefkovits J. Clinical impact of a high-sensitivity troponin assay introduction on patients presenting to the emergency department. Emerg Med Australas. 2016;28:273-8.

109. Perera M, Aggarwal L, Scott IA, Logan B. Received care compared to ADP-guided care of patients admitted to hospital with chest pain of possible cardiac origin. Int J Gen Med. 2018;11:345-51.

110. Pettersson A, Ljung L, Johansson C, Heilborn U, Jernberg T, Frick M, et al. Experiences of a One-hour Algorithm in Chest Pain Patients With a Nonelevated Troponin T at Presentation. Crit Pathw Cardiol. 2018;17:6-12.

111. Pickering JW, Young JM, George P, Aldous S, Cullen L, Greenslade JH, et al. The utility of presentation and 4-hour high sensitivity troponin I to rule-out acute myocardial infarction in the emergency department. Clin Biochem. 2015;48:1219-24.

112. Pickering JW, Greenslade JH, Cullen L, Flaws D, Parsonage W, Aldous S, et al. Assessment of the European Society of Cardiology 0-Hour/1-Hour Algorithm to Rule-Out and Rule-In Acute Myocardial Infarction. Circulation. 2016;134:1532-41.

113. Pickering JW, Flaws D, Smith SW, Greenslade J, Cullen L, Parsonage W, et al. A Risk Assessment Score and Initial High-sensitivity Troponin Combine to Identify Low Risk of Acute Myocardial Infarction in the Emergency Department. Acad Emerg Med. 2018;25:434-43.

114. Poldervaart JM, Reitsma JB, Koffijberg H, Backus BE, Six AJ, Doevendans PA, et al. Impact of using the HEART score in chest pain patients at the emergency department: a stepped wedge, cluster randomized trial. Eur Heart J. 2016;37:593‐4.

115. Poldervaart JM, Reitsma JB, Backus BE, Koffijberg H, Veldkamp RF, Ten Haaf ME, et al. Effect of Using the HEART Score in Patients with Chest Pain in the Emergency Department: A Stepped-Wedge, Cluster Randomized Trial. Ann Intern Med. 2017;166:689-97.

116. Rabbani LE, Biviano AB, Bergmann SR, Tenenbaum J, Sullivan J, Hurley E, et al. Design of a Comprehensive Chest Pain Initiative in an Academic Urban Hospital. Crit Pathw Cardiol. 2003;2:113-7.

117. Rao MPR, Panduranga P, Al-Mukhaini M, Sulaiman K, Al-Jufaili M. Predictive value of a 4-hour accelerated diagnostic protocol in patients with suspected ischemic chest pain presenting to an emergency department. Oman Med J. 2012;27:207-11.

118. Reichlin T, Schindler C, Drexler B, Twerenbold R, Reiter M, Zellweger C, et al. One-hour rule-out and rule-in of acute myocardial infarction using high-sensitivity cardiac troponin T. Arch Intern Med. 2012;172:1211-8.

119. Reichlin T, Cullen L, Parsonage WA, Greenslade J, Twerenbold R, Moehring B, et al. Two-hour algorithm for triage toward rule-out and rule-in of acute myocardial infarction using high-sensitivity cardiac troponin T. Am J Med. 2015;128:369-79.e4.

120. Reichlin T, Twerenbold R, Wildi K, Gimenez MR, Bergsma N, Haaf P, et al. Prospective validation of a 1-hour algorithm to rule-out and rule-in acute myocardial infarction using a high-sensitivity cardiac troponin T assay. CMAJ. 2015;187:E243-52.

121. Richards G, Sen G, Halliday A, Thomas S, Stevenson E, Gillings M, et al. Reducing chest pain admissions using a 1 hour high-sensitivity Troponin-T pathway. Heart. 2017;103:A50‐1.

122. Rottger E, de Vries-Spithoven S, Reitsma JB, Limburg A, van Ofwegen-Hanekamp CEE, Hoes AW, et al. Safety of a 1-hour Rule-out High-sensitive Troponin T Protocol in Patients with Chest Pain at the Emergency Department. Crit Pathw Cardiol. 2017;16:129-34.

123. Ruangsomboon O, Thirawattanasoot N, Chakorn T, Limsuwat C, Monsomboon A, Praphruetkit N, et al. The utility of the 1-hour high-sensitivity cardiac troponin T algorithm compared with and combined with five early rule-out scores in high-acuity chest pain emergency patients. Int J Cardiol. 2021;322:23-8.

124. Sanchis J, Garcia-Blas S, Mainar L, Mollar A, Abellan L, Ventura S, et al. High-sensitivity versus conventional troponin for management and prognosis assessment of patients with acute chest pain. Heart. 2014;100:1591-6.

125. Sandoval Y, Smith SW, Shah AS V, Anand A, Chapman AR, Love SA, et al. Rapid Rule-Out of Acute Myocardial Injury Using a Single High-Sensitivity Cardiac Troponin I Measurement. Clin Chem. 2017;63:369-76.

126. Sandoval Y, Smith SW, Thordsen SE, Bruen CA, Carlson MD, Dodd KW, et al. Diagnostic Performance of High Sensitivity Compared with Contemporary Cardiac Troponin I for the Diagnosis of Acute Myocardial Infarction. Clin Chem. 2017;63:1594-604.

127. Sandoval Y, Smith SW, Schulz K, Sexter A, Apple FS. Comparison of 0/3-Hour Rapid Rule-Out Strategies Using High-Sensitivity Cardiac Troponin I in a US Emergency Department. Circ Cardiovasc Qual Outcomes. 2020;13:e006565.

128. Schonemann-Lund M, Schoos MM, Iversen K, Hansen SI, Thode J, Clemmensen P, et al. Retrospective Evaluation of Two Fast-track Strategies to Rule Out Acute Coronary Syndrome in a Real-life Chest Pain Population. J Emerg Med. 2015;49:833-42.

129. Shah AS V, Anand A, Sandoval Y, Lee KK, Smith SW, Adamson PD, et al. High-sensitivity cardiac troponin I at presentation in patients with suspected acute coronary syndrome: a cohort study. Lancet. 2015;386:2481-8.

130. Shah AS V, Anand A, Strachan FE, Ferry A V, Lee KK, Chapman AR, et al. High-sensitivity troponin in the evaluation of patients with suspected acute coronary syndrome: a stepped-wedge, cluster-randomised controlled trial. Lancet. 2018;392:919-28.

131. Sharp AL, Baecker AS, Shen E, Redberg R, Lee M-S, Ferencik M, et al. Effect of a HEART Care Pathway on Chest Pain Management Within an Integrated Health System. Ann Emerg Med. 2019;74:171-80.

132. Shiozaki M, Inoue K, Suwa S, Lee C-C, Chikata Y, Ishiura J, et al. Utility of the 0-hour/1-hour high-sensitivity cardiac troponin T algorithm in Asian patients with suspected non-ST elevation myocardial infarction. Int J Cardiol. 2017;249:32-5.

133. Shiozaki M, Inoue K, Suwa S, Lee C-C, Chiang S-J, Sato A, et al. Implementing the European Society of Cardiology 0-h/1-h algorithm in patients presenting very early after chest pain. Int J Cardiol. 2020;320:1-6.

134. Shortt C, Xie F, Whitlock R, Ma J, Clayton N, Sherbino J, et al. Economic Considerations of Early Rule-In/Rule-Out Algorithms for The Diagnosis of Myocardial Infarction in The Emergency Department Using Cardiac Troponin and Glycemic Biomarkers. Clin Chem. 2017;63:593-602.

135. Skoien W, Cullen L, Ashover S, Sippel J, Bettens V, Parsonage W. Outcomes of the Queensland accelerated chest pain risk evaluation (ACRE) project. Heart Lung Circ. 2015;24:S159.

136. Slagman A, von Recum J, Mockel M, Holert F, Meyer Zum Buschenfelde D, Muller C, et al. Diagnostic performance of a high-sensitive troponin T assay and a troponin T point of care assay in the clinical routine of an Emergency Department: A clinical cohort study. Int J Cardiol. 2017;230:454-60.

137. Smulowitz PB, Dizitzer Y, Tadiri S, Thibodeau L, Jagminas L, Novack V. Impact of implementation of the HEART pathway using an electronic clinical decision support tool in a community hospital setting. Am J Emerg Med. 2018;36:408-13.

138. Snavely AC, Hendley N, Stopyra JP, Lenoir KM, Wells BJ, Herrington DM, et al. Sex and race differences in safety and effectiveness of the HEART pathway accelerated diagnostic protocol for acute chest pain. Am Heart J. 2021;232:125-36.

139. Stopyra JP, Miller CD, Hiestand BC, Lefebvre CW, Nicks BA, Cline DM, et al. Chest Pain Risk Stratification: A Comparison of the 2-Hour Accelerated Diagnostic Protocol (ADAPT) and the HEART Pathway. Crit Pathw Cardiol. 2016;15:46-9.

140. Stopyra JP, Snavely AC, Lenoir KM, Wells BJ, Herrington DM, Hiestand BC, et al. HEART Pathway Implementation Safely Reduces Hospitalizations at One Year in Patients With Acute Chest Pain. Ann Emerg Med. 2020;76:555-65.

141. Stopyra J, Snavely AC, Hiestand B, Wells BJ, Lenoir KM, Herrington D, et al. Comparison of accelerated diagnostic pathways for acute chest pain risk stratification. Heart. 2020;106:977-84.

142. Stopyra JP, Snavely AC, Ashburn NP, Nelson RD, McMurray EL, Hunt MR, et al. EMS blood collection from patients with acute chest pain reduces emergency department length of stay. Am J Emerg Med. 2021;47:248-52.

143. Storrow AB, Christenson RH, Nowak RM, Diercks DB, Singer AJ, Wu AHB, et al. Diagnostic performance of cardiac Troponin I for early rule-in and rule-out of acute myocardial infarction: Results of a prospective multicenter trial. Clin Biochem. 2015;48:254-259.

144. Stoyanov KM, Hund H, Biener M, Gandowitz J, Riedle C, Loehr J, et al. Effects of implementation of the ESC 0/1-hour algorithm on efficiacy and safety of discharge after rule-out in a consecutive cohort of patients with suspected acute coronary syndrome-RAPID-CPU study. Eur Heart J. 2019;40:1941.

145. Stoyanov KM, Biener M, Gandowitz J, Riedle C, Lohr J, Mueller-Hennessen M, et al. RAPID-CPU: a prospective study on implementation of the ESC 0/1-hour algorithm and safety of discharge after rule-out of myocardial infarction. Eur Heart J Acute Cardiovasc Care. 2019; http://dx.doi.org/10.1177/2048872619861911

146. Stoyanov KM, Biener M, Hund H, Mueller-Hennessen M, Vafaie M, Katus HA, et al. Effects of crowding in the emergency department on the diagnosis and management of suspected acute coronary syndrome using rapid algorithms: an observational study. BMJ Open. 2020;10:e041757.

147. Stoyanov KM, Hund H, Biener M, Gandowitz J, Riedle C, Lohr J, et al. RAPID-CPU: a prospective study on implementation of the ESC 0/1-hour algorithm and safety of discharge after rule-out of myocardial infarction. Eur Heart J Acute Cardiovasc Care. 2020;9:39-51.

148. Tan JWC, Sahlen AO, Yeo KK, Chin WLC, Tan HJG, Gao F, et al. Performance of cardiac troponins within the HEART score in predicting major adverse cardiac events at the emergency department. Am J Emerg Med. 2019;38(8):1560-7.

149. Than M, Cullen L, Reid CM, Lim SH, Aldous S, Ardagh MW, et al. A 2-h diagnostic protocol to assess patients with chest pain symptoms in the Asia-Pacific region (ASPECT): a prospective observational validation study. Lancet. 2011;377:1077-1084.

150. Than M. An RCT of a 2-hour protocol to identify low-risk patients presenting with possible acute coronary syndrome suitable for safe early discharge. J Amer Coll Cardiol. 2012;19:743.

151. Than M, Aldous S, Lord SJ, Goodacre S, Frampton CMA, Troughton R, et al. A 2-hour diagnostic protocol for possible cardiac chest pain in the emergency department: a randomized clinical trial. JAMA Intern Med. 2014;174:51-8.

152. Than M, Flaws D, Sanders S, Doust J, Glasziou P, Kline J, et al. Development and validation of the Emergency Department Assessment of Chest pain Score and 2 h accelerated diagnostic protocol. Emerg Med Australas. 2014;26:34-44.

153. Than MP, Pickering JW, Aldous SJ, Cullen L, Frampton CMA, Peacock WF, et al. Effectiveness of EDACS Versus ADAPT Accelerated Diagnostic Pathways for Chest Pain: A Pragmatic Randomized Controlled Trial Embedded Within Practice. Ann Emerg Med. 2016;68:93-102.e1.

154. Thet EM, Murphy JJ, Crilley JG. P2712 Integration of new CENTAUR high-sensitivity Troponin I assay with HEART score chest pain pathway to maximise early discharge from emergency department. Eur Heart J. 2019;40:ehz748-1029.

155. Thiruganasambandamoorthy V, Stiell IG, Chaudry H, Mukarram M, Booth RA, Toarta C, et al. Use of conventional cardiac troponin assay for diagnosis of non-ST-elevation myocardial infarction: “The Ottawa Troponin Pathway”. PLoS One. 2020;15:e0226892.

156. Twerenbold R, Reichlin T, Reiter M, Haaf P, M RG, Wildi K, et al. Impact of the clinical introduction of high-sensitivity cardiac troponin T assay on rates of coronary angiographies and exercise stress tests in acute chest pain-insights from an international trial. Eur Heart J. 2014;35:983-4.

157. Twerenbold R, Boeddinghaus J, Nestelberger T, Rubini Gimenez M, Puelacher C, Miro O, et al. One-hour rule-out and rule-in of acute myocardial infarction using a novel high-sensitivity cardiac troponin I assay. Eur Heart J. 2017;38:1006.

158. Twerenbold R, Boeddinghaus J, Nestelberger T, Rubini Gimenez M, Puelacher C, Badertscher P, et al. One-hour rule-out and rule-in of acute myocardial infarction using a novel ultra-sensitive cardiac troponin I assay. Eur Heart J. 2018;39:1368‐9.

159. Twerenbold R, Neumann JT, Sorensen NA, Ojeda F, Karakas M, Boeddinghaus J, et al. Prospective Validation of the 0/1-h Algorithm for Early Diagnosis of Myocardial Infarction. J Am Coll Cardiol. 2018;72:620-32.

160. Twerenbold R, Costabel JP, Nestelberger T, Campos R, Wussler D, Arbucci R, et al. Outcome of Applying the ESC 0/1-hour Algorithm in Patients With Suspected Myocardial Infarction. J Am Coll Cardiol. 2019;74:483-94.

161. Whitehead S, Ford C, Gama R, Willmer K. High sensitivity cardiac troponin i at admission enables early safe discharge, reduces hospital stay and prevents unnecessary hospital admissions. Clin Chem Lab Med. 2017;55:S62.

162. Wildi K, Nelles B, Twerenbold R, Rubini Gimenez M, Reichlin T, Singeisen H, et al. Safety and efficacy of the 0 h/3 h protocol for rapid rule out of myocardial infarction. Am Heart J. 2016;181:16-25.

163. Wildi K, Cullen L, Twerenbold R, Greenslade JH, Parsonage W, Boeddinghaus J, et al. Direct Comparison of 2 Rule-Out Strategies for Acute Myocardial Infarction: 2-h Accelerated Diagnostic Protocol vs 2-h Algorithm. Clin Chem. 2017;63:1227-1236.

164. Yang SM, Chan CH, Chan TN. HEART pathway and Emergency Department Assessment of Chest Pain Score-Accelerated Diagnostic Protocol application in a local emergency department of Hong Kong: An external prospective validation study. Hong Kong J Emerg Med. 2020;27:30-8.

165. Yau AA, Nguyendo LT, Lockett LL, Michaud E. The HEART Pathway and Hospital Cost Savings. Crit Pathw Cardiol. 2017;16:126-8.

166. Yip TPY, Pascoe HM, Lane SE. Impact of high-sensitivity cardiac troponin I assays on patients presenting to an emergency department with suspected acute coronary syndrome. Med J Aust. 2014;201:158-61.
