# Supplementary material for: Effectiveness of accelerated diagnostic protocols for reducing emergency department length of stay in patients presenting with chest pain: A systematic review and meta-analysis
Source: PLoS One. 2024 Oct 22;19(10):e0309767. doi: 10.1371/journal.pone.0309767 (PMC11495623; doi:10.1371/journal.pone.0309767)
Supplement: S5 File — (DOCX) [file pone.0309767.s011.docx]

| **S5 File. Risk of bias domains and overall ratings for the included observational studies (Newcastle−Ottawa Scores)** | | | | | | | | |
| --- | --- | --- | --- | --- | --- | --- | --- | --- |
| **Study** | **Domain 1** | **Domain 2** | **Domain 3** | **NOS** | **Domain 1** | **Domain 2** | **Domain 3** | **Overall** |
| Al Marashi 2020 [1] | 4 | 2 | 0 | 6 | Low | Low | High | Low |
| Allen 2018 [2] | 4 | 2 | 2 | 8 | Low | Low | Low | Low |
| Barnes 2021 [3] | 4 | 1 | 3 | 8 | Low | Some Concerns | Low | Low |
| Bevins 2022 [4] | 4 | 2 | 1 | 7 | Low | Low | Some Concerns | Some Concerns |
| Buttinger 2019 [5] | 4 | 1 | 1 | 6 | Low | Some Concerns | Some Concerns | Low |
| Crowder 2015 [6] | 4 | 1 | 3 | 8 | Low | Some Concerns | Low | Low |
| Ford 2021 [7] | 4 | 2 | 3 | 9 | Low | Low | Low | Low |
| Furmaga 2021 [8] | 4 | 0 | 1 | 5 | Low | High | Some Concerns | Some Concerns |
| Ganguli 2021 [9] | 4 | 2 | 3 | 9 | Low | Low | Low | Low |
| Greenslade 2020 [10] | 4 | 1 | 1 | 6 | Low | Some Concerns | Some Concerns | Some Concerns |
| Hill 2023 [11] | 4 | 1 | 3 | 8 | Low | Some Concerns | Low | Low |
| Hughes 2023 [12] | 4 | 2 | 1 | 7 | Low | Low | Some Concerns | Low |
| Ljung 2019 [13] | 4 | 2 | 2 | 8 | Low | Low | Low | Low |
| Mahler 2018 [14] | 4 | 2 | 3 | 9 | Low | Low | Low | Low |
| Mohmed 2021 [15] | 2 | 2 | 1 | 5 | Some Concerns | Low | Some Concerns | Some Concerns |
| Mountain 2016 [16] | 4 | 2 | 3 | 9 | Low | Low | Low | Low |
| Mumma 2020 [17] | 4 | 2 | 1 | 7 | Low | Low | Some Concerns | Low |
| Mungai 2020 [18] | 3 | 2 | 2 | 7 | Low | Low | Low | Low |
| Ola 2021 [19] | 4 | 2 | 3 | 9 | Low | Low | Low | Low |
| Parsonage 2017 [20] | 4 | 2 | 3 | 9 | Low | Low | Low | Low |
| Phillips 2023 [21] | 4 | 1 | 3 | 8 | Low | Some Concerns | Low | Low |
| Randolph 2018 [22] | 4 | 1 | 0 | 5 | Low | Some Concerns | High | High |
| Rowe 2023 [23] | 4 | 2 | 1 | 7 | Low | Low | Some Concerns | Low |
| Ruangsomboon 2018 [24] | 4 | 1 | 1 | 6 | Low | Some Concerns | Some Concerns | Some Concerns |
| Suh 2022 [25 | 4 | 2 | 2 | 8 | Low | Low | Low | Low |
| Than 2018 [26] | 4 | 2 | 3 | 9 | Low | Low | Low | Low |
| Than 2021 [27] | 3 | 0 | 2 | 5 | Low | High | Low | Low |
| Trent 2022 [28] | 4 | 1 | 3 | 8 | Low | Some Concerns | Low | Low |
| Twerenbold 2016 [29] | 4 | 1 | 1 | 6 | Low | Some Concerns | Some Concerns | Some Concerns |
| Tyner 2023 [30] | 4 | 2 | 2 | 8 | Low | Low | Low | Low |
| VanAssche 2023 [31] | 4 | 2 | 1 | 7 | Low | Low | Some Concerns | Low |
| Vigen 2020 [32] | 4 | 2 | 2 | 8 | Low | Low | Low | Low |

**References**

1. Al Marashi H, Bhuiyan K, Sankhla V, Khan L, Carroll M, Symington L, et al. 503 Implementation of a High Sensitivity Troponin Assay and Overnight Rapid Emergency Department (ED) Discharge of Intermediate Risk Patients: Impact on the ED Length of Stay. Heart Lung Circ. 2020;29: S264.

2. Allen BR, Simpson GG, Zeinali I, Freitas JT, Chapa JJ, Rawson LJ, et al. Incorporation of the HEART Score Into a Low-risk Chest Pain Pathway to Safely Decrease Admissions. Crit Pathw Cardiol. 2018;17(4):184-90.

3. Barnes C, Fatovich DM, Macdonald SPJ, Alcock RF, Spiro JR, Briffa TG, et al. Single high-sensitivity troponin levels to assess patients with potential acute coronary syndromes. Heart. 2021;107(9):721-7.

4. Bevins NJ, Chae H, Hubbard JA, Castillo EM, Tolia VM, Daniels LB, Fitzgerald RL. Emergency Department Management of Chest Pain With a High-Sensitivity Troponin-Enabled 0/1-Hour Rule-Out Algorithm. Am J Clin Pathol. 2022;157(5):774-80.

5. Buttinger N, White D, Shurlock J, Blows L, Dooley M. Comparing the Length of Stay, Efficacy and Safety of a New 1 Hour "Rule-out" Pathway to Standard Care for Patients Presenting to the Emergency Departments with a Suspected Acute Coronary Syndrome at Brighton and Sussex University Hospitals NHS Trust. Heart. 2019;105:A125-A6.

6. Crowder KR, Jones TD, Lang ES, Wang DM, Clark SM, Innes GD, et al. The impact of high-sensitivity troponin implementation on hospital operations and patient outcomes in 3 tertiary care centers. Am J Emerg Med. 2015;33(12):1790-4.

7. Ford JS, Chaco E, Tancredi DJ, Mumma BE. Impact of high-sensitivity cardiac troponin implementation on emergency department length of stay, testing, admissions, and diagnoses. Am J Emerg Med. 2021;45:54-60.

8. Furmaga J, McDonald SA, Hall HM, Muthukumar A, Willett K, Basit M, Diercks DB. Impact of High-sensitivity Troponin Testing on Operational Characteristics of an Urban Emergency Department. Acad Emerg Med. 2021;28(1):114-66.

9. Ganguli I, Cui J, Thakore N, Orav EJ, Januzzi JL, Baugh CW, et al. Downstream Cascades of Care Following High-Sensitivity Troponin Test Implementation. J Am Coll Cardiol. 2021;77(25):3171-9.

10. Greenslade JH, Ho A, Hawkins T, Parsonage W, Crilly J, Cullen L. Examining the translational success of an initiative to accelerate the assessment of chest pain for patients in an Australian emergency department: a pre-post study. BMC Health Serv Res. 2020;20(1):419.

11. Hill J, Yang E, Lefebvre D, Doran S, Raizman J, Tsui A, Rowe BH. Effect of the introduction of a high sensitivity troponin I and associated diagnostic protocol on emergency department length of stay: a single site retrospective cohort study. Canadian Journal of Emergency Medicine. 2023;25(1):S74.

12. Hughes AEO, Forbriger A, May AM, Scott MG, Char D, Farnsworth CW. Implementation of high-sensitivity troponin with a rapid diagnostic algorithm reduces emergency department length of stay for discharged patients. Clin Biochem. 2023;116:87-93.

13. Ljung L, Lindahl B, Eggers KM, Frick M, Linder R, Lofmark HB, et al. A Rule-Out Strategy Based on High-Sensitivity Troponin and HEART Score Reduces Hospital Admissions. Ann Emerg Med. 2019;73(5):491-9.

14. Mahler SA, Lenoir KM, Wells BJ, Burke GL, Duncan PW, Case LD, et al. Safely Identifying Emergency Department Patients With Acute Chest Pain for Early Discharge. Circulation. 2018;138(22):2456-68.

15. Mohmed S, Campbell M, Batouskaya K, Obeidat M, Khand A. Implementation of the ESC 0/3hour accelerated diagnostic protocol, utilising high sensitive troponin T, for suspected acute coronary syndrome:a study on clinical effectiveness involving 3016 patients. Eur Heart J Acute Cardiovasc Care. 2021;10: i100.

16. Mountain D, Ercleve T, Allely P, McQuillan B, Yamen E, Beilby J, et al. REACTED - Reducing Acute Chest pain Time in the ED: A prospective pre-/post-interventional cohort study, stratifying risk using early cardiac multi-markers, probably increases discharges safely. Emerg Med Australas. 2016;28(4):383-90.

17. Mumma BE, Dang R, Adams EJ, Tran N, Amsterdam E, Tancredi DJ. High-sensitivity cardiac troponin and emergency department length of stay: A before and after study. . Academic Emergency Medicine. 2020;27(S1):S47.

18. Mungai E, Hamilton BK, Burns D. Comparison of High-Sensitivity Troponin T Assay to Conventional Troponin T Assay for Rule Out of Acute Coronary Syndrome in the Emergency Department. Adv Emerg Nurs J. 2020;42(4):304-14.

19. Ola O, Akula A, De Michieli L, Dworak M, Crockford E, Lobo R, et al. Clinical Impact of High-Sensitivity Cardiac Troponin T Implementation in the Community. J Am Coll Cardiol. 2021;77(25):3160-70.

20. Parsonage WA, Milburn T, Ashover S, Skoien W, Greenslade JH, McCormack L, Cullen L. Implementing change: evaluating the Accelerated Chest pain Risk Evaluation (ACRE) project. Med J Aust. 2017;207(5):201-5.

21. Phillips D, Shelton D, Verma A, Liu S, Yeung V, Cheng I. Impact of a high sensitivity troponin accelerated diagnostic protocol on the safety and emergency department length of stay of chest pain in an academic tertiary hospital: a quality improvement study. CJEM. 2023;25(11):909-19.

22. Randolph FT, Slovis BH, London KS, Chang AM, Aini M, Weitz HH, et al. Impact of High Sensitivity Troponins of Discharge Rates of Patients Presenting to the Emergency Department With Chest Pain. Annals of Emergency Medicine. 2018;72(4):S2-S3.

23. Rowe BH, Yang E, Doran S, Graham M, Van Diepen S, Raizman J, Tsui A. Impact of a rapid high-sensitivity troponin protocol on length of stay for adult patients presenting with chest pain to a tertiary care emergency department. Canadian Journal of Emergency Medicine. 2023;25(1):S50-S1.

24. Ruangsomboon O, Mekavuthikul P, Chakorn T, Monsomboon A, Prapruetkit N, Surabenjawong U, et al. The feasibility of the 1-h high-sensitivity cardiac troponin T algorithm to rule-in and rule-out acute myocardial infarction in Thai emergency patients: an observational study. Int J Emerg Med. 2018;11(1):43.

25. Suh EH, Tichter AM, Ranard LS, Amaranto A, Chang BC, Huynh PA, et al. Impact of a rapid high-sensitivity troponin pathway on patient flow in an urban emergency department. J Am Coll Emerg Physicians Open. 2022;3(3):e12739.

26. Than MP, Pickering JW, Dryden JM, Lord SJ, Aitken SA, Aldous SJ, et al. ICare-ACS (Improving Care Processes for Patients With Suspected Acute Coronary Syndrome): A Study of Cross-System Implementation of a National Clinical Pathway. Circulation. 2018;137(4):354-63.

27. Than MP, Pickering JW, Adamson P, Clendon T, Florkowski CM, Lainchbury J, et al. Reducing Patient Risk and Enhancing Care Through the Development and Implementation of a New Chest Pain Pathway, Expedited by and for the COVID-19 Era. EJIFCC. 2021;32(1):27-40.

28. Trent S, Gravitz S, Krantz M, Haukoos J. 324 Effect of High-sensitivity Troponin on Emergency Department Length of Stay for Patients Evaluated for Acute Coronary Syndrome. Annals of Emergency Medicine. 2022;80(4):S138.

29. Twerenbold R, Jaeger C, Rubini Gimenez M, Wildi K, Reichlin T, Nestelberger T, et al. Impact of high-sensitivity cardiac troponin on use of coronary angiography, cardiac stress testing, and time to discharge in suspected acute myocardial infarction. Eur Heart J. 2016;37(44):3324-32.

30. Tyner RJ, Whittington MD, Patterson VP, Ho M, Pincus S, Wiler JL, Michael SS. Differences in cardiac testing resource utilization using two different risk stratification schemes. Am J Emerg Med. 2023;65:179-84.

31. Van Assche L, Peeters B, Vorlat A, Monsieurs K, Heidbuchel H, Claeys MJ. Safety and effectiveness of the short (0-1h) high sensitive troponin protocol in real-life practice. Acta Cardiol. 2023;78(8):937-44.

32. Vigen R, Diercks DB, Hashim IA, Pandey A, Zhong L, Kutscher P, et al. Association of a Novel Protocol for Rapid Exclusion of Myocardial Infarction With Resource Use in a US Safety Net Hospital. JAMA Netw Open. 2020;3(4):e203359.
