# Supplementary material for: Effectiveness of accelerated diagnostic protocols for reducing emergency department length of stay in patients presenting with chest pain: A systematic review and meta-analysis
Source: PLoS One. 2024 Oct 22;19(10):e0309767. doi: 10.1371/journal.pone.0309767 (PMC11495623; doi:10.1371/journal.pone.0309767)
Supplement: S6 File — (DOCX) [file pone.0309767.s012.docx]

| **S6 File. Risk of bias domains and overall ratings for the included randomized controlled studies (assessed using the Cochrane Risk of Bias tool)** | | | |
| --- | --- | --- | --- |
| **Study** | **Source of bias** | **Authors’ judgment** | **Support for judgment** |
| Anand 2021 [1] | 1. Random sequence generation (selection bias) | Low risk | The researchers embedded the screening tool into the patient record to ensure that they prospectively enrolled consecutive patients in whom the attending clinician suspected ACS. This minimized the risk of selection bias, ensuring that they did not limit their findings to low-risk patients or those presenting within working hours. |
|  | 2. Allocation concealment (selection bias) | Unclear risk | It is unclear whether allocation was concealed based on the narrative. |
|  | 3. Blinding of participants and personnel (performance bias)  All outcomes | Low risk | The intervention was implemented at the hospital level; thus, the authors did not seek individual patient consent. This reduced the risk of a Hawthorne effect, where effectiveness is exaggerated through direct observation of clinical care by researchers. The primary and secondary safety outcomes of myocardial infarction or cardiac death at 30 days and 1 year was adjudicated by a panel blinded to the study phase. Two physicians independently reviewed all clinical information, and discordant diagnoses were resolved by a third reviewer. |
|  | 4. Blinding of outcome assessment (detection bias)  All outcomes | Low risk | The intervention was implemented at the hospital level; thus, the authors did not seek individual patient consent. The primary and secondary safety outcomes of myocardial infarction or cardiac death at 30 days and 1 year was adjudicated by a panel blinded to the study phase. Two physicians independently reviewed all clinical information, and discordant diagnoses were resolved by a third reviewer. |
|  | 5. Incomplete outcome data (attrition bias)  All outcomes | Low risk | Following randomization, only 64 out of 31492 patients who migrated out of Scotland were excluded from the analysis. |
|  | 6. Selective reporting (reporting bias) | Low risk | There does not appear to have been any selective reporting for either arm of randomization. |
|  | 7. Other bias | Low risk | Study appears to be free of other sources of bias |
| Carlton 2020 [2] | 1. Random sequence generation (selection bias) | Low risk | The Peninsula Clinical Trials Unit, in conjunction with the study statistician, provided web-based randomization, stratified by center and minimized by age and gender. |
|  | 2. Allocation concealment (selection bias) | Low risk | Once the randomization process was complete, the computer screen indicated to treating clinicians which diagnostic strategy to follow, including details of the local rule out strategy for control participants as a reminder for site staff. A print-out of the allocation generated by the randomization website was taken and filed in the participant’s ED records |
|  | 3. Blinding of participants and personnel (performance bias)  All outcomes | High risk | This was an open study. Participants’ study allocations will only be blinded to those performing central review of data for the assessment of MACE and the statistician analyzing the results. |
|  | 4. Blinding of outcome assessment (detection bias)  All outcomes | Low risk | This was an open study. Participants’ study allocations will only be blinded to those performing central review of data for the assessment of MACE and the statistician analyzing the results. |
|  | 5. Incomplete outcome data (attrition bias)  All outcomes | Low risk | No patients were lost to follow up after randomization as per the CONSORT diagram. Data for 2 patients in the usual care group and 7 patients in the LoDED group were missing. |
|  | 6. Selective reporting (reporting bias) | Low risk | There does not appear to have been any selective reporting for both arms of the trial. |
|  | 7. Other bias | Low risk | Study appears to be free of other sources of bias. |
| Chew 2019 [3] | 1. Random sequence generation (selection bias) | Low risk | The randomization schedule was generated by an independent statistician and implemented by a sealed envelope process. |
|  | 2. Allocation concealment (selection bias) | Low risk | Randomization to troponin reporting formats occurred before any troponin assays are undertaken. |
|  | 3. Blinding of participants and personnel (performance bias)  All outcomes | Unclear risk | No indications from the study and protocol on measures used to blind trial participants and researchers from knowledge of which intervention a participant received. |
|  | 4. Blinding of outcome assessment (detection bias)  All outcomes | Low risk | hs-TnT elevations underwent blinded adjudication for MI to confirm event time. |
|  | 5. Incomplete outcome data (attrition bias)  All outcomes | Low risk | The attrition rate (2.66%) was lower than expected in the study protocol (3%). All attritions and exclusions were reported for the main outcomes, as were the reasons. There were no re-inclusions in the analysis. |
|  | 6. Selective reporting (reporting bias) | Low risk | There does not appear to have been any selective reporting for the study population with suspected ACS. Primary and secondary outcomes were reported for both the overall ITT group and patients with troponin concentrations <29 ng/L, as were outcomes for both arms of randomization. |
|  | 7. Other bias | Low risk | Study appears to be free of other sources of bias. |
| Lambrakis 2021 [4] | 1. Random sequence generation (selection bias) | Low risk | Randomization was performed using 4-block permutations and stratified by participating facility |
|  | 2. Allocation concealment (selection bias) | Low risk | The design of the study would not permit allocation concealment. |
|  | 3. Blinding of participants and personnel (performance bias)  All outcomes | High risk | At the ED, masking was only done in the standard 0/3 h arm in which the treating physician was blinded to Troponin T values <29 ng/L. |
|  | 4. Blinding of outcome assessment (detection bias)  All outcomes | Low risk | Index admissions and subsequent events were adjudicated by at least 2 independent cardiologists with disagreements assessed by a third senior cardiologist, all of whom were blinded to the randomization arm, and each primary end point event was reviewed at a clinical events committee meeting. |
|  | 5. Incomplete outcome data (attrition bias)  All outcomes | Low risk | Overall, only 108 participants withdrew and the data for 12-month follow-up was available for 3270 participants. |
|  | 6. Selective reporting (reporting bias) | Low risk | Outcomes do not appear to have been selectively reported. Outcomes were pre-declared in the protocol and retrieved through systematic interrogation of statewide hospital and administrative data and utilization of data linkage methods. |
|  | 7. Other bias | Low risk | Study appears to be free of other sources of bias. |
| Miller 2022 [5] | 1. Random sequence generation (selection bias) | Unclear risk | Unclear from the abstract. |
|  | 2. Allocation concealment (selection bias) | Unclear risk | Unclear from the abstract. |
|  | 3. Blinding of participants and personnel (performance bias)  All outcomes | Unclear risk | Unclear from the abstract. |
|  | 4. Blinding of outcome assessment (detection bias)  All outcomes | Unclear risk | Unclear from the abstract. |
|  | 5. Incomplete outcome data (attrition bias)  All outcomes | Low risk | No evidence of attrition for the primary and secondary outcomes |
|  | 6. Selective reporting (reporting bias) | Low risk | No evidence of selective reporting in the abstract. |
|  | 7. Other bias | Low risk | Study appears to be free of other sources of bias. |

**References**

1. Anand A, Lee KK, Chapman AR, Ferry AV, Adamson PD, Strachan FE, et al. High-Sensitivity Cardiac Troponin on Presentation to Rule Out Myocardial Infarction: A Stepped-Wedge Cluster Randomized Controlled Trial. Circulation. 2021;143(23):2214-24.

2. Carlton EW, Ingram J, Taylor H, Glynn J, Kandiyali R, Campbell S, et al. Limit of detection of troponin discharge strategy versus usual care: randomised controlled trial. Heart. 2020;106(20):1586-94.

3. Chew DP, Lambrakis K, Blyth A, Seshadri A, Edmonds MJR, Briffa T, et al. A Randomized Trial of a 1-Hour Troponin T Protocol in Suspected Acute Coronary Syndromes: The Rapid Assessment of Possible Acute Coronary Syndrome in the Emergency Department With High-Sensitivity Troponin T Study (RAPID-TnT). Circulation. 2019;140(19):1543-56.

4. Lambrakis K, Papendick C, French JK, Quinn S, Blyth A, Seshadri A, et al. Late Outcomes of the RAPID-TnT Randomized Controlled Trial: 0/1-Hour High-Sensitivity Troponin T Protocol in Suspected ACS. Circulation. 2021;144(2):113-25.

5. Miller J, Nassereddine H, Jennings K, Connor S, Modi S, Yan J, et al. Economic Impact: A Cluster Randomized Trial of a Rapid, High-Sensitivity Cardiac Troponin I Protocol. Annals of Emergency Medicine. 2022;80(4):S45.
