# Supplementary material for: Effectiveness of accelerated diagnostic protocols for reducing emergency department length of stay in patients presenting with chest pain: A systematic review and meta-analysis
Source: PLoS One. 2024 Oct 22;19(10):e0309767. doi: 10.1371/journal.pone.0309767 (PMC11495623; doi:10.1371/journal.pone.0309767)
Supplement: S8 File — (PDF) [file pone.0309767.s014.pdf]

The effectiveness of accelerated diagnostic protocols for reducing emergency department length of stay in patients presenting with chest pain: a systematic review

Citation

Jesse Hill, Esther Yang, Elizabeth Dennett, Albert Tsui, Sean Van Diepen, Brian Rowe. The effectiveness of accelerated diagnostic protocols for reducing emergency department length of stay in patients presenting with chest pain: a systematic review. PROSPERO 2021 CRD42021249679 Available from:
https://www.crd.york.ac.uk/prospero/display_record.php?ID=CRD42021249679

Review question

Are accelerated diagnostic protocols for adult patients presenting with chest pain to an emergency department effective in reducing emergency department lengths of stay, compared to standard care?

Searches

We will identify relevant studies via systematic searches of bibliographic databases including: MEDLINE, EMBASE, CENTRAL, Scopus, LILACS, CINAHL, and Proquest dissertation abstracts.

A grey literature search will be conducted as follows:

a) A search of clinical trial registries (Cochrane Central Register of controlled trials, controlled-trials.com and ClinicalTrials.gov) will be completed to identify recent and ongoing studies.

b) Backward and forward Scopus searches of included studies will be completed at the end of the search to identify additional relevant studies.

c) Google Scholar web search.

d) Hand searches of the most recent emergency medicine conference abstracts associated with Canadian (Canadian Association of Emergency Physicians-CAEP; Canadian Journal of Emergency Medicine-CJEM), US (American College of Emergency Physicians-ACEP; Annals of Emergency Medicine) and international (Society for Academic Emergency Medicine-SAEM; Academic Emergency Medicine-AEM) emergency medicine research meetings to identify recently completed but not yet published studies.

e) In addition, we will search bibliographies from known reviews and text for additional citations.

Types of study to be included

Clinical trials (RCTs and NRCTs), controlled cohort studies (retrospective and prospective), and before-after studies are eligible to be included in this review.

Condition or domain being studied

Patients with presentations to the emergency department with a chief complaint of chest pain are common. Chest pain is associated with considerable health care resource use, consultations to specialists, hospitalization and a high rate of seven-day ED relapse requiring hospital admission and resulting in poor outcomes. Due to both its prevalence and potential severity, considerable research has focused on standardizing assessment and risk stratification for chest pain

Participants/population

Studies assessing adults (> 18 years) seen in an emergency department (or equivalent) are eligible for inclusion. No restrictions will be set on the study population assessed in a study.

Intervention(s), exposure(s)

Any accelerated diagnostic protocol designed to decrease the time between initial troponin blood draw and eventual disposition for chest pain.

Comparator(s)/control

Standard diagnostic protocols for evaluating chest pain in the emergency department, typically involving a 6-hour serial troponin. The studies must provide a well-matched control population.

Context

Studies assessing ED operational outcomes after the implementation of an accelerated diagnostic protocol for chest pain will be eligible to be included in this review.

Main outcome(s)

The primary outcome of interest will be ED length of stay

Additional outcome(s)

1. Proportion of patients left without being seen (LWBS)

2. Proportion of patients with a Cardiology consult;

3. Proportion of patients requiring admission;

4. Proportion of patients with 72 hour return to ED/admission;

5. Proportion of patients with major adverse cardiac events (MACE) defined as the composite of total death, MI, stroke , hospitalization because of HF, and revascularization, including percutaneous coronary intervention, and coronary artery bypass graft.

Data extraction (selection and coding)

Study identification will involve a two-step process using two independent reviewers. First, from the title, abstract, or descriptors, we will independently review literature searches to identify potentially relevant studies for full review. Second, from the full text, using specific criteria, we will independently select studies for inclusion in this review. Disagreement will be resolved by third party adjudication; reasons for exclusion will be documented. Data will be extracted onto standardized forms that will include information on study characteristics, patient population, diversion strategy description, and outcomes of interest. The data will be extracted via one independent reviewer, and verified for accuracy by another reviewer. Any disagreements that cannot be resolved via consensus will be resolved via third party mediation

Risk of bias (quality) assessment

The risk of bias of RCT/CCT’s will be assessed using the Cochrane Risk of Bias (RoB) tool. The risk of bias of before-after studies will be assessed using the Before-after quality assessment (BAQA) checklist. Finally, the quality of observational cohorts will be assessed using the Ottawa New-Castle assessment scale. Two reviewers will independently

evaluate the methodological quality of the studies and disagreements will be discussed and resolved with a third-party mediator

Strategy for data synthesis

Studies will be pooled if they represented similar populations, outcomes, and designs, and the research team has judged that heterogeneity was sufficiently low. Statistical analysis of the data will be completed via RevMan (Update Software, Oxford, Version 5.3). For continuous outcomes individual trials results will be reported as mean differences (MD) or standardized mean differences (SMD) will be pooled as weighted mean differences (WMD) with 95% CI using a random effects model. The weights given to each study in the pooled analysis will be based on the Mantel-Haenszel method. If the heterogeneity among the studies, or insufficient outcome reporting, prohibits the pooling of the data, then a descriptive analysis of the data will be completed.

Analysis of subgroups or subsets

Assuming sufficient heterogeneity and outcome reporting allow for a meta-analysis, several subgroup/sensitivity analyses are planned. The studies will be sub-grouped based on study design (RCT/ CCT vs. cohort vs. before/after). Planned subgroup analysis of the primary outcome (ED LOS) will include specific ADP used (i.e. HEART vs EDACS). Planned sensitivity analyses will include study quality (in which high risk of bias studies will be excluded).

Contact details for further information

Jesse Hill

jlh1@ualberta.ca

Organisational affiliation of the review

University of Alberta

Review team members and their organisational affiliations

Dr Jesse Hill. University of Alberta

Esther Yang. University of Alberta

Ms Elizabeth Dennett. University of Alberta

Dr Albert Tsui. University of Alberta

Dr Sean Van Diepen. University of Alberta

Dr Brian Rowe. University of Alberta

Type and method of review

Intervention, Systematic review

Anticipated or actual start date

15 April 2021

Anticipated completion date

01 June 2021

Funding sources/sponsors

Canadian Institutes of Health Research (CIHR)

Conflicts of interest

Language

English

Country

Canada

Stage of review

Review Ongoing

Subject index terms status

Subject indexing assigned by CRD

Subject index terms

Chest Pain; Emergency Service, Hospital; Humans; Length of Stay

Date of registration in PROSPERO

12 May 2021

Date of first submission

17 April 2021

Stage of review at time of this submission

**Stage**	**Started**	**Completed**
Preliminary searches	Yes	No
Piloting of the study selection process	No	No
Formal screening of search results against eligibility criteria	No	No
Data extraction	No	No
Risk of bias (quality) assessment	No	No
Data analysis	No	No

The record owner confirms that the information they have supplied for this submission is accurate and complete and they

understand that deliberate provision of inaccurate information or omission of data may be construed as scientific misconduct.

The record owner confirms that they will update the status of the review when it is completed and will add publication details in due course.

Versions

12 May 2021
