# Supplementary material for: Effectiveness of accelerated diagnostic protocols for reducing emergency department length of stay in patients presenting with chest pain: A systematic review and meta-analysis
Source: PLoS One. 2024 Oct 22;19(10):e0309767. doi: 10.1371/journal.pone.0309767 (PMC11495623; doi:10.1371/journal.pone.0309767)
Supplement: Attachment — Submitted filename: Reviewers Comments.docx [file pone.0309767.s015.docx]

Thank you for the opportunity to review the manuscript entitled “*Effectiveness of accelerated diagnostic protocols (ADPs) for reducing emergency department length of stay in patients presenting with chest pain: A systematic review***”**. The authors performed a novel systematic review on the usefulness of ADPs on the main outcome of length of stay (LOS) in the emergency department (ED). Meta-analysis was done but the authors genuinely did not reflect it in the title due to the high rate of heterogeneity. They fairly considered potential confounders and have them adjusted in meta-regression.

# Previous systematic reviews and meta-analyses have addressed the efficacy of the HEART score for detecting MACE, which was a secondary outcome in this study. How this study will affect clinical practice? Nowadays, most clinicians use ADPs. The results regarding hsTn vs. Tn in terms of LOS seem to be more practical.

There are some comments regarding this article:

# Major points:

# *Abstract*:

# It has been concluded that ADPs can reduce LOS but did not change admission rates although in the result section it is mentioned that ADPs significantly changed the admission rate. This needs to be clarified.

# *Methods:*

# In this systematic review, not a single test (criteria) but studies with various ADPs were included such as HEART pathway, IMPACT, COVID path, ESC algorithm, ADAPT, STEACS and their data were pooled. Although most of the ADPs consist of the same items, still there are some different items for example: syncope and CK-MB in IMPACT. This can result in a high risk of bias, which should be addressed and mentioned in the limitation section.

# Moreover, 16/37 studies, including 3/5 RCTs, did not state which ADP they used. The meta-analysis could be done excluding these studies regardless of the overall ROB score.

# The definition for LOS was different among various studies: for instance,

Ref 21: the definition for the ED LOS: from patients’ arrival to ED disposition

Ref 45: from patients’ arrival to ED departure

They should cautiously pool these data and mention this issue in the limitation section.

***Results:***

- For secondary objectives, clinically practical data of admission days and MACE can be elaborated. For example, what was the time range for changes in admission rate?
- I found the S1 supplementary table very useful as well even though they were secondary outcomes, they present practical outcomes for clinicians.

***Conclusions:***

- The authors fairly mentioned the limitations of this study and accordingly, the conclusion should be revised and interpreted cautiously since the heterogeneity was high (>99% for the primary outcome). It is recommended to mainly focus on descriptive reports of the study results and not to optimistically rely on meta-analysis.

**Minor Point:**

- This sentence needs to be clarified about “changes”, Line 200-201:

“Three studies reported changes specific to discharged patients, while the remaining studies reported changes for all patients.” For example, were discharged patients younger or without past medical history, etc? What did the authors mean by specific changes for all the patients?
