# Supplementary material for: Effectiveness of accelerated diagnostic protocols for reducing emergency department length of stay in patients presenting with chest pain: A systematic review and meta-analysis
Source: PLoS One. 2024 Oct 22;19(10):e0309767. doi: 10.1371/journal.pone.0309767 (PMC11495623; doi:10.1371/journal.pone.0309767)
Supplement: Attachment — Submitted filename: b_ADP_Responses 3.7.docx [file pone.0309767.s016.docx]

**Manuscript ID: PONE-D-24-12460**

**Response to reviewers Table**

**July 22, 2024**

| **Comment** | **Our adjustments** |
| --- | --- |
| **Academic editor** | |
| 1. When submitting your revision, we need you to address these additional requirements.   Please ensure that your manuscript meets PLOS ONE’s style requirements, including those for file naming. | We have ensured that the revised manuscript meets the style requirements of *PLOS ONE* as indicated in the templates, including those for file naming. |
| 2. Please identify your study as “systematic review and meta-analysis” in the title of your manuscript. | We have amended the title of the revised manuscript to:  *Effectiveness of accelerated diagnostic protocols for reducing emergency department length of stay in patients presenting with chest pain: A systematic review* ***and meta-analysis.*** |
| 3. Please also include the statement “There was no additional external funding received for this study.” in your updated Funding Statement. Please include your amended Funding Statement within your cover letter. We will change the online submission form on your behalf. | We have updated our funding statement in the cover letter to the following:  *Dr. Jesse Hill has no funding sources to report. Dr. Brian Rowe’s research is supported by a Scientific Director’s Grant (SOP 168483) from the Canadian Institutes of Health Research (CIHR, Ottawa, Ontario). Ms. Esther Yang is supported by the Emergency Medicine Research Group (EMeRG) in the Department of Emergency Medicine, University of Alberta.* ***There was no additional external funding received for this study.*** *The funders take no responsibility for the conduct, analyses, and interpretation of these results.* |
| 4. This work reports the utility of accelerated diagnostic protocols for chest pain in improving operational efficiency. While the primary outcome is not patient-centered, and thus, limit the value of the conclusions drawn to daily practice, documenting the role of APDs in process improvement is a worthwhile endeavor and I commend the authors for undertaking this task masterfully. | We thank the Academic Editor for taking the time to assess our manuscript. Your comments and recommendations are greatly appreciated. We agree that there is value to describing the literature on process improvement and appreciate the opportunity to expand on this within this revision and resubmission. |
| 5. *Abstract:* Well written. Rationale clearly stated. | Thank you; we appreciate your comment. |
| 6. Line 41: Please state the follow-up time for the MACE assessment here. | The follow-up was variable and determined by the study authors; however, most studies reported 30-day MACE outcomes. We have amended the highlighted statement to read:  Abstract, page 3  *Secondary outcomes included the proportion of patients admitted and the proportion with* ***30-day*** *major adverse cardiac events (MACE).* |
| 7. Lines 44–47: Is difficult to understand. Please consider re-writing for clarity. | We have amended the highlighted statement to read:  Abstract, page 3  ***Emergency departments with longer baseline*** *ED LOS* ***showed*** *significantly* ***larger reductions*** *in LOS* ***after ADP implementation****.* ***This observed association persisted*** *after adjusting for both the change in serial Tn measurement interval and transition from conventional Tn assay to a hs-Tn assay (β = -0.26; 95% CI, -0.43 to -0.10).* |
| 8. *Introduction:* Well written. | Thank you for this supportive comment. |
| 9. *Methods:* Admission proportion: Is there a baseline admission rate? | Thank you for this request for clarification. Admission proportion is a more appropriate term to describe hospitalization than admission rate, because rates require a time variable (e.g., number of admissions/time frame [e.g., year]) which was not reported in any studies.  All outcomes (including admission proportion, which was reported in 24 out of 37 included studies) had values reported for both the Pre/Before (baseline) and Post/After groups. This information is summarized in Supplementary Table 1. |
| *Results:* |  |
| 10. Table 1: please define what the “Before”, “After” columns mean. | For consistency with the headings for subgroups shown in Table 2 and the S1 Table, we have changed the headers of the “Before” and “After” columns in Table 1 to “Pre” and “Post”, respectively. In the footnotes to each table, we have indicated that these indicate values or estimates reported before and after ADP implementation, respectively. |
| 11. Lines 200–201: Which changes are being referred to here? ED LOS? | Your interpretation is correct. We have corrected the highlighted statement to:  Results, page 11  *Three studies reported* ***characteristics*** ***and*** *changes* ***in ED LOS*** *specific to discharged patients, while the remaining studies reported* ***characteristics and*** *changes* ***in ED LOS*** *for all enrolled/included patients.* |
| 12. Table 2: edit the title to include reference to before and after ADP implementation. | We have amended the title of Table 2 as follows:  *Summary of troponin types used, delta troponin measurement intervals, and ED LOS reported* ***before and after ADP implementation*** *among the included studies.* |
| 13. Table 2: what is the unit of the Tn interval measure? | The Tn interval measure reflects the time (in hours) between the first (baseline) and second Tn serial measurements. This has been clarified in Table 2 as follows:  *Tn interval* ***(hours)*** |
| *Discussion:* |  |
| 14. Line 288: LOS probably belongs somewhere in the sentence. | Your interpretation is correct. The highlighted statement has been amended to:  Discussion, page 17  *One consideration for* ***the*** *minimal reductions* ***in LOS seen*** *in these EDs may be due to wait times rather than protocol efficiency.* |
| 15. Given the large heterogeneity in the studies, perhaps a thematic review of the observational studies to tease out the best parts of each observational study may have been more beneficial instead of a meta-analysis. | Thank you for your thoughtful comment. Our decision to present our findings within a meta-analytic framework was driven by the primary objective of our systematic review, which was to quantitatively assess the operational and clinical outcomes of ADPs implemented in patients with suspected cardiac chest pain. Despite the acknowledged heterogeneity, which is common in systematic reviews of facility-level interventions, meta-analysis offers a rigorous method to derive summary estimates and explore potential sources of variability through subgroup and sensitivity analyses.  Also, a thematic review was not within the scope of funding and our expertise. We have thus taken measures to assess and address the heterogeneity within our meta-analysis and indicated cautious interpretation of the pooled results where necessary. |
| 16. Line 359–360: If the claim that pooling was valid based on the similarities in the patient population, could the heterogeneity be at the level of which ADP was implemented? Sub-group analysis by ADP type would likely have more clinical value. | This is an excellent question. Unfortunately, this was also not our experience. As shown in Table 2, a large number of studies did not report the specific type of ADP used (*k* = 16). We had the following specified ADPs: ADAPT (*k* = 1), COVID Pathway (*k* = 1), ESC algorithm (*k* = 4), HEART (*k* = 10), IMPACT (k = 1), multiple ADPs (*k* = 2), and other ADPs (*k* = 2). In our stratified analysis (not considering ADAPT, COVID Pathway, and IMPACT which had one study each), the heterogeneity was still high within ADP types: ESC algorithm (*I*^2^ = 100.0%), HEART pathway (*I*^2^ = 98.5%), multiple ADPs (*I*^2^ = 98.7%), and *I*^2^ = 99.2% for the ‘other’ ADP category. Thus, we are of the view that a sub-group analysis by ADP type would still not be beneficial from the clinical standpoint. |
| 17. So, it is the specific algorithm not troponin assay type, which seems to drive the reduced LOS. Is this statement correct? | This is another excellent question. Further to your point no. 16 above, this was not the case. Stratifying the meta-regression analysis looking for drivers of the reduction in ED LOS for specific ADP type could lead to potentially spurious results, considering that there are multiple ‘unspecific’ categories. This is why we resorted to using change in serial Tn measurement and transition from conventional Tn to hs-Tn assay as ADP components in our adjusted meta-regression. A post-hoc meta-regression considering studies that outright declared HEART ADP vs. those that did not failed to identify a significant difference between the subgroups. This has been mentioned in the text as follows:  Discussion, page 21  ***A sensitivity analysis comparing studies that reported versus did not report the ADP type, however, showed very similar and overlapping effect estimates, indicating that the absence of specific ADP information did not substantially impact the overall findings.*** |
| 18. Please comment on the performance of ADPs in general against clinical gestalt in terms of ED LOS. In my experience, a convincing story with a high Tn usually leads to an admission. So, the value of the ADP perhaps lies in those scenarios where the story is not clear. The clinical utility of this study may have been enhanced if the authors focused on those kinds of ADPs which addressed this scenario. | In light of your suggestion, we have commented on this briefly:  Discussion, page 17  ***These findings are important because while clinical gestalt remains a critical component of decision-making in patients with typical clinical presentations and high troponin levels, ADPs complement clinical gestalt by providing a reliable, evidence-based framework for managing patients with chest pain. This framework mitigates the variability associated with gestalt alone, ensuring that most patients are discharged safely and efficiently. The overall results provide direction for clinicians and administrators and the additional findings below provide opportunities for further refinement and implementation research.*** |
| *Conclusion:* |  |
| 19. The first sentence was not a stated objective of the study. Please edit. | We have revised the highlighted sentence to:  Conclusions, page 22  *This systematic review examined the* ***operational and clinical outcomes*** *of* ***ADPs implemented*** *for patients presenting* ***to EDs*** *with* ***suspected cardiac*** *chest pain.* |
| 20. Line 372: Please edit this sentence. Since observational studies were included in the LOS estimates, caution should be exercised when drawing causative conclusions. | We agree with the Reviewer’s comment and have amended the text as follows:  Conclusions, pages 21−22  *Our findings* ***showed that implementing*** *an ADP* ***may significantly*** *reduce ED LOS and should be considered by hospitals or healthcare entities searching for strategies to improve operational efficiency.* ***This decrease in LOS*** *was even seen in the absence of any change in the* ***type of*** *Tn assay.* |
| **Reviewer #1** | |
| 1. The authors performed a novel systematic review on the usefulness of ADPs on the main outcome of length of stay (LOS) in the emergency department (ED). Meta-analysis was done but the authors genuinely did not reflect it in the title due to the high rate of heterogeneity. They fairly considered potential confounders and have them adjusted in meta-regression.   Previous systematic reviews and meta-analyses have addressed the efficacy of HEART score for detecting MACE, which was a secondary outcome in this study. How this study will affect clinical practice? Nowadays, most clinicians use ADPs. The results regarding hsTn vs. Tn in terms of LOS seems to be more practical. | We thank Reviewer 1 for taking the time to assess our manuscript. Your comments and recommendations are greatly appreciated. |
| *Abstract:*   1. It has been concluded that ADPs can reduce LOS but did not change admission rates although in the result section it is mentioned that ADPs significantly changed the admission rate. This needs to be clarified. | The risk ratio (RR = 0.56; 95% CI, 0.49−0.64) we previously reported was our estimate from the values presented by Ljung et al. (2019) which showed the largest reduction in admissions following ADP implementation. We have deleted the study-level result and reported the pooled estimate of RR = 0.84 (95% CI, 0.79−0.89). We have also attached the forest plot for that analysis as our new Supplementary Figure 3. Subsequently, we have revised the text as follows:  Abstract, page 3  *The decrease in LOS* ***occurred alongside reductions in*** *hospital admissions****, while not increasing*** *MACE.*  Conclusions, page 22  *Moreover, this decrease in LOS was associated with meaningful reductions in hospital admissions,* ***without an increase*** *in subsequent adverse events (such as MACE).* |
| *Methods:* |  |
| 2. In this systematic review, not a single test (criteria) but studies with various ADPs were included such as HEART pathway, IMPACT, COVID path, ESC algorithm, ADAPT, STEACS and their data were pooled. Although most of the ADPs consist of same items, still there are some different items for example: syncope and CK-MB in IMPACT. This can result in a high risk of bias, which should be addressed and mentioned in the limitation section. | We however agree that the heterogeneity of ADPs included in our analysis represents a limitation, and we have now explicitly acknowledged this as follows:  Strengths and limitations, page 21:  ***The inclusion of studies utilizing different ADPs introduces additional heterogeneity due to the variability in the criteria used. While most ADPs share common components, differences such as the inclusion of syncope and CK-MB in the IMPACT protocol may influence outcomes.*** |
| 3. Moreover, 16/37 studies, including 3/5 RCTs, did not state which ADP they used. The meta-analysis could be done excluding these studies regardless of the overall ROB score. | Thank you for highlighting this important consideration. In a post-hoc subgroup analysis comparing studies that reported the specific ADP used to those that did not, the subgroup estimates were very similar and their confidence intervals overlapped significantly. This suggests that the presence or absence of specific ADP information would not substantially alter the pooled effect estimates we presented. We have revised the text as follows to reflect this:  Strengths and limitations, page 21  ***Again, a number of included studies did not specify the ADP used. A sensitivity analysis comparing studies that reported versus did not report the ADP type, however, showed very similar and overlapping effect estimates, indicating that the absence of specific ADP information did not substantially impact the overall findings.*** |
| 4. The definition for LOS was different among various studies: for instance,   Ref 21: the definition for the ED LOS: from patients’ arrival to ED disposition  Ref 45: from patients’ arrival to ED departure  They should cautiously pool these data and mention this issue in the limitation section. | Thank you for raising this important point. We carefully reviewed the studies and summarized their definitions in Supplementary Table 2. Some authors, such as Bevins et al. (2022) considered ED LOS as the time from ED arrival to ED disposition, but reported separate values for discharged vs. admitted patients. For such papers, we compared the ED LOS for discharged patients since they comprised the larger majority and the cohort(s) were mostly low to intermediate-risk patients. Our post-hoc subgroup analysis showed that the individual estimates for the subgroups of studies that reported ED LOS as the time from ED arrival to ED disposition vs. ED discharge were very similar and did not statistically significantly differ. We have highlighted this among our limitations, as follows:  Strengths and limitations, page 21  ***In particular, the definition of ED LOS varied among the included studies (S2 Table), with a large majority defining it as the time from ED arrival to either discharge or disposition. Two studies did not use either definition; Suh et al. [38] defined ED LOS as the time from first clinician provider evaluation to disposition decision time (provider-to-disposition time), while Anand et al. [46] defined it as the time from ED arrival (presentation) to hospital discharge.*** |
| *Results:* |  |
| 5. For secondary objectives, clinically practical data of admission days and MACE can be elaborated. For example, what was the time range for changes in admission rate? | The time range for changes in admission proportion, influenced by the adoption of ADPs, varied across studies, primarily due to differences in study designs, patient populations, and healthcare settings. Generally, the studies did not provide other information on admission days and MACE apart from the proportions. Thus, we are unable to speak to this request. |
| 6. I found the S1 supplementary table very useful as well even though they were secondary outcomes, they present practical outcomes for clinicians. | Thank you; we appreciate you pointing this out. |
| *Conclusions:* |  |
| 7. The authors fairly mentioned the limitations of this study and accordingly, the conclusion should be revised and interpreted cautiously since the heterogeneity was high (>99% for the primary outcome). It is recommended to mainly focus on descriptive reports of the study results and not to optimistically rely on meta-analysis. | We agree with the Reviewer. In the revised Conclusions, we have refrained from overemphasizing the results of the meta-analyses, as follows:  Conclusions, pages 21−22  *This systematic review examined the* ***operational and clinical outcomes*** *of* ***ADPs implemented*** *for patients presenting* ***to EDs*** *with* ***suspected cardiac*** *chest pain. Our findings* ***showed*** *that* ***implementing*** *an ADP* ***may significantly reduce*** *ED LOS and should be considered by hospitals or healthcare entities searching for strategies to improve operational efficiency.* ***This decrease in*** *LOS* ***was even*** *seen in the absence of any change in* ***the type of*** *Tn assay. Moreover, this decrease in LOS* ***showed*** *meaningful* ***reductions in*** *hospital admissions****, without an increase in*** *subsequent adverse events (such as MACE). The observed benefits also translated across multiple countries and health regions. Further research should evaluate the optimal combination of Tn measurement interval in combination with specific ADPs.* |
| *Minor point:* |  |
| 8. This sentence need to be clarified about “changes”, Line 200−201:   “Three studies reported changes specific to discharged patients, while the remaining studies reported changes for all patients.” For example, were discharged patients younger or without past medical history, etc? What did authors mean by specific changes for all the patients? | In the highlighted sentence, we were specifically referring to changes in ED LOS as a primary outcome of interest. We have revised it as follows:  Results, page 11  *Three studies reported* ***characteristics*** ***and*** *changes* ***in ED LOS*** *specific to discharged patients, while the remaining studies reported* ***characteristics and*** *changes* ***in ED LOS*** *for all patients.* |
| **Reviewer #2** | |
| 1. The article titled “Effectiveness of Accelerated Diagnostic Protocols for Reducing Emergency Department Length of Stay in Patients Presenting with Chest Pain: A Systematic Review” offers a comprehensive analysis of the impact of accelerated diagnostic protocols (ADPs) on the efficiency and operational metrics for evaluating suspected cardiac chest pain in emergency departments. | We thank the reviewer for taking the time to assess our work and for the positive comments. |
| 2. As noted by the authors, previous studies have established the safety of accelerated chest pain protocols, showing similar proportions of admissions and Major Adverse Cardiac Events (MACE) compared to traditional methods. In this study, the authors investigate how effectively ADPs improve patient flow through emergency departments by analyzing their impact on operational metrics. The review encompasses 37 articles involving over 404,566 patients across various countries and healthcare settings, enhancing the generalizability of the findings. The study adhered to PRISMA guidelines and was registered in PROSPERO. Risk of bias was assessed using established tools, and data extraction was performed independently by multiple reviewers. | This is an accurate reflection of the study methods and results. |
| 3. While it is intuitive to expect these protocols to improve patient flow, the authors' key findings are significant: ED Length of Stay (LOS) showed substantial reductions, especially in departments with higher baseline LOS; implementing ADPs did not lead to an increase in adverse cardiac events, thereby maintaining patient safety; and there was a decrease in admission rates after implementation, indicating a reduction in unnecessary admissions without compromising patient safety. The authors also address and discuss the limitations of their work, including heterogeneity in study design and reporting variability. | This is an accurate reflection of the study findings. |
| 4. This study is a valuable contribution to emergency medicine, and I recommend acceptance without further revisions. | The authors would like to thank the reviewer for these positive comments. |
